# Digital Microfluidics-Driven Cell-Free Protein Synthesis Platform Reveals Expression and Stability Determinants for Phytoglobins and Cysteine-to-Alanine Substituted Variants

**DOI:** 10.3390/antiox14111317

**Published:** 2025-10-31

**Authors:** Leonard Groth, Leif Bülow

**Affiliations:** Division of Pure and Applied Biochemistry, Lund University, 221 00 Lund, Sweden; leif.bulow@tbiokem.lth.se

**Keywords:** phytoglobin, heme protein, cell-free protein synthesis, digital microfluidics, solubility tag screen, redox environment, expression determinants, cysteine

## Abstract

Heme proteins are central to metabolism and stress responses but remain challenging to express recombinantly due to cytotoxicity and folding constraints. Phytoglobins (Pgbs) exemplify these difficulties, as expression protocols often fail to translate across protein species. Here, we used a cell-free protein synthesis (CFPS) platform powered by digital microfluidics to screen expression determinants for sugar beet Pgb 1.2 (BvPgb 1.2), its C86A variant, and three of eight newly identified oat Pgbs (AsPgbs), including their cysteine-to-alanine substituted variants. Benchmarking with multiple solubility tags and cell-free blends revealed protein- and variant-specific preferences, with alanine substitutions frequently improving expression and purification yields. Oxidative additives such as glutathione disulfide, alone or combined with protein disulfide isomerase, consistently enhanced production, underscoring the importance of redox environments for Pgb stability. Two selected variants were scaled up and yielded putative soluble apo-form proteins. The results highlight how CFPS enables rapid, parallelized identification of expression requirements while uncovering the role of conserved cysteines and redox conditions in Pgb biogenesis.

## 1. Introduction

Heme proteins such as cytochromes, catalases, and globins are central to energy metabolism, signaling, and stress responses across all domains of life [1,2,3,4]. Despite their biological importance, producing novel heme proteins in heterologous hosts remains challenging. The cytotoxicity of heme, coupled with the metabolic burden of its biosynthesis, often results in poor yields or insoluble products [5]. Globins are particularly challenging in this regard, as unfolded apoglobins are thought to have a high rate of aggregation [6]. Globin expression needs to synchronize protein synthesis with heme synthesis to avoid inclusion bodies [7], metabolic disruptions, and premature cell death, whilst at the same time obtaining meaningful yields [5]. Expression conditions therefore need to be finely balanced, yet methods optimized for one heme protein do not necessarily translate directly to others [8].

Phytoglobins (Pgbs), plant globins induced through various forms of oxidative stress, exemplify this problem [9,10,11,12]. Pgbs are involved in redox control [13], nitric oxide (NO)-driven metabolism [14,15,16,17], and NO and radical oxygen species (ROS) scavenging [18]. Previous work on BvPgb 1.2, a class 1 Pgb from sugar beet (*Beta vulgaris*), enabled detailed characterization of both the recombinant wild-type (rWT, PDB: 7ZOS) protein and cysteine-to-alanine substituted variant (C86A, PDB: 7Z1U) [19,20,21,22,23,24,25]. In this paper we report eight novel oat (*Avena sativa*) Pgbs, distributed across three distinct orthogroups covering two class 1 Pgb (AsPgb 1.1–1.3, and AsPgb 1.4–1.6) and one class 3 Pgb (AsPgb 3.1–3.2). Despite predicted structural similarities between the class 1 AsPgbs and BvPgb 1.2 as shown here, attempts to express the AsPgbs through similar methods have been met with limited success. Here, we test how different solubility (SOL) tags, cysteine-to-alanine substitutions, and various additives individually affect Pgb expression and stability, hypothesizing that each parameter contributes distinct constraints on yield and solubility.

To address these challenges and bypass the many trial-and-error cycles associated with developing workable protocols for intractable proteins like Pgbs, we turned to a cell-free protein synthesis (CFPS) platform capable of screening multiple expression conditions in parallel. The platform is run on digital microfluidics cartridges (DMCs) configured to screen proteins-of-interest (POIs) fused with SOL tags N-terminally linked by 3C protease target sites. A total of 24 different constructs are accommodated on the DMC in one of either 3 × 8, 4 × 6, or 6 × 4 POI × SOL layouts. These constructs are in turn screened against eight different cell-free blends (CFBs), yielding a total of 192 unique POIs expressed under 64, 48, or 32 distinct CFPS conditions, depending on the POI × SOL layout.

The DMC can manipulate, mix, merge, and split individual droplets through electrowetting [26], which underlies the mechanistic foundation of the CFPS platform. POI synthesis and homogeneity is monitored through bimolecular fluorescence complementation (BiFC) [27,28], which is induced when the C-terminally fused GFP11 tag (DET) is mixed with its parental GFP 1-10 molecule [28]. Measured expression yields serve as selection criteria for downstream streptactin-based purification. This is likewise facilitated by a Strep tag II (STREP) placed after the DET tag. The translational machinery (e.g., polymerases, ribosomes) of the CFPS platform is derived from *Escherichia coli* lysate, and together with e.g., energy components, nucleotides, and amino acids (aa), constitute the cell-free core reagent (CFCR) of each CFB.

In this work we screened common chaperones by preparing three CFBs by supplementation of either thioredoxin reductase (TRXB1) [29], DnaK mix [30], or glutathione disulfide (GSSG) mixed protein disulfide isomerase (PDI/GSSG) [31]. A CFB supplemented with GSSG alone was used to screen the effect of PDI. Common cofactors associated with improving protein folding, stability, and activity were also screened in two CFBs on exploratory grounds by adding zinc chloride (Zn^2+^) [32] or a cofactor mix of NAD, acetyl-CoA, FAD, SAM, and PLP [33]. To screen the intrinsic impact of the SOL tag, a CFB with 3C protease was included. As a comparative control, one CFB was prepared with a standard buffer. To probe heme insertion, all used CFBs were also supplemented with a custom NaOH-dissolved hemin solution.

In this paper, we first benchmarked BvPgb 1.2, C86A, and a H-NOX variant on the 3 × 8 DMC design. The benchmarking probed seven commonly used SOL tags (P17 [34], CUSF [35], FH8 [36], TRX [37], ZZ [38], SUMO [39], and SNUT [40]) against the untagged POIs (–).

Informed by the benchmark screen, a 6 × 4 DMC design was opted for the AsPgbs where P17, CUSF, and FH8 were probed against their untagged counterparts. This design doubles the number of POIs screened but halves the number of unique expression conditions and purification selections. The 6 × 4 screen included one representative of each AsPgb orthogroup (AsPgb 1.1, 1.5, and 3.1) along their alanine-substituted variant (C70A, C84A, and C161A, respectively). These variants were included due to prior work indicating conflicting roles for the conserved cysteine residue on structural integrity, aggregation, and dimerization [19] as compared to other class 1 Pgbs [41]. Exploring the impact of this residue across multiple Pgbs in CFPS systems is of interest, as the stability of nascent apoglobins is critical for successful holoprotein formation [6].

One POI from each DMC run was selected for scale-up in 200 µL reactions, namely CUSF-BvPgb 1.2 and P17-AsPgb 1.5. The oligomeric state and thermal stability of the scaled-up POIs were assessed by mass photometry (MP) and nano differential scanning fluorimetry (nanoDSF). These analyses aimed to probe if differences in these biophysical properties could account for the limited success of applying the established recombinant expression protocol from BvPgb 1.2 to the AsPgb.

## 2. Materials and Methods

All water used (ddH_2_O) was biology grade with a resistivity of 18.2 MΩ·cm and total organic content of less than five parts per billion.

### 2.1. In Silico Analysis of Putative AsPgb Candidates

To identify putative AsPgb candidates, the Sequenceserver v2.0.0 [9] tool was used to identify putative AsPgb candidates by performing iterative BLASTP (E-value < 1.0 × 10^–5^) searches with different queries against oat cv. Sang v1.1 and OT3098 v1 PepsiCo [10] protein databases. First, we queried all members of the plant globin family that abide by rules ARBA: ARBA00007609 and UniRule RuleBase: RU000625 against the oat cv. Sang v1.1 proteins to capture potentially non-annotated AsPgbs. From here we continued using more curated protein model queries from barley (*Hordeum vulgare*), thale cress (*Arabidopsis thaliana*), and rice (*Oryza sativa*). Protein sequences for the putative AsPgbs were retrieved from the oat reference genome [42], and each member of corresponding orthogroups were aligned against each other using Clustal Omega [43]. Tissue expression patterns were also identified within the oat reference genome [42].

### 2.2. Structural Comparison of BvPgb and Predicted Putative AsPgb Structures

Sequence similarity between AsPgb 1.1 and 1.5 against BvPgb 1.2 were determined through BLASTp [44]. The protein sequences for AsPgb 1.1, 1.5, and 3.1 were submitted to ColabFold [45] for structure prediction. The resulting model was structurally aligned to the BvPgb 1.2 7ZOS.pdb file in UCSF ChimeraX [46]. Structural matchmaking was performed using a backbone (bb) chain pairing strategy to guide the alignment of protein structures. Sequence–structure alignments were generated using the Needleman–Wunsch global alignment algorithm with the BLOSUM-62 similarity matrix as the scoring basis. Secondary structure information was incorporated into the alignment with a weighting fraction of 0.3, ensuring that both sequence similarity and structural context contributed to the scoring.

Gap penalties were applied differentially depending on the alignment context. Gap opening costs were set to 18 for both helix and strand regions and 6 for other regions, while a uniform gap extension penalty of 1 was used. Iterative refinement was applied with a cutoff of two iterations, allowing the alignment to converge under both sequence and structural constraints.

### 2.3. Construction of eGene™ Constructs

Synthesized DNA fragments corresponding to the genes of interest (GOIs, i.e., BvPgb 1.2, C86A, AsPgb 1.1, C70A, AsPgb 1.5, C84A, AsPgb 3.1, and C161A) pre-adapted with 3C and TEV sequences on 5′ and 3′ ends, respectively, were designed using the eProtein Discovery^TM^ Cloud Software v5.1.0 (Nuclera, Cambridge, UK) purchased as gBlocks^TM^ (IDT, Coralville, IA, USA) and normalized to 2 nM stocks. GOI encoding eGene™ constructs were made using the Nuclera eGene Prep Solubility Tag Kit (Nuclera, Cambridge, UK). PCR reactions of 60 µL contained 19 µL ddH_2_O; 30 µL 2× high-fidelity Phusion™ Plus PCR Master Mixes (Thermo Fisher Scientific, Waltham, MA, USA); 1 µL of either 2 nM DNA template (GOI or positive control template) or ddH_2_O for the negative control; and 10 µL of eGene Primer Mix for either P17, FH8, HSUMO3, TRX, SNUT, CUSF, ZZ, or untagged variants. AsPgbs encoding eGenes were constructed as either P17, FH8, CUSF, or untagged variants.

Reactions were assembled on ice and briefly centrifuged before amplification run. The run consisted of 1 cycle preincubation at 98 °C, 30 s followed by 27 cycles of 98 °C, 10 s denaturation; 60 °C, 20 s annealing; and 72 °C, 30 s/kb elongation (rounded up), before a final elongation at 72 °C for 2 min. During the first 5–10 cycles, overlap extension between the 3C and TEV sequences yielded full-length assemblies, which were then exponentially amplified by the universal terminal primers. PCR products were purified by column-based methods and eluted in the eGene Elution Buffer (50 µL). Amplified DNAs were then quantified by NanoDrop (Thermo Fisher Scientific, Waltham, MA, USA) and normalized to 5 nM. Quality control was performed by agarose gel electrophoresis (1% *w*/*v*) to verify the expected product size and purity.

### 2.4. Solubility Tag Cell-Free Protein Synthesis and Purification Screen

The CFPS DMCs (eProtein Discovery^TM^ cartridge, Nuclera, Cambridge, UK) were supplied with a cartridge reagent kit and strep beads for this experiment. The cartridge consists of a total of 52 different ports, labeled A1–A8 (topmost); B1–B8 (top); C1–C8 (bottom); H1-H8 (bottom-most); A10–H10 (left); A12–H12 (right); and four corner ports clockwise oriented as X1 (top left), X2 (top right), X3 (bottom right), and X4 (bottom left). All reagents for ports A1–A8, B1–B8, C1–C8, and H1–H8 were loaded at 3 µL and reagents for A10–H10 and A12–H12 at 12 µL.

For each experiment, 5 µL of each eGene construct was transferred into the DNA designated wells of the DMC transfer plate. For the 3 × 8 design, each eGene construct for BvPgb 1.2 and C86A was added into ports A1–A8 and B1–B8, respectively, with eGenes ordered by SOL tags as P17, CUSF, FH8, TRX, ZZ, SUMO, SNUT, and none. For the 6 × 4 design, AsPgb1.1, AsPgb1.1 C70A, AsPgb1.5, AsPgb1.5 C84A, AsPgb3.1, and AsPgb3.1 C161A were added to ports A1–A4, A5–A8, B1–B4, B5–B8, C1–C4, and C5–C8, respectively, with SOL tag order being P17, CUSF, FH8, and none.

Eight CFBs were prepared by combining 16 µL of CFCR, 2 µL of customized hemin additive, and 2 µL of eight different additives, including standard buffer (HEPES buffer pH 7.5)**,** PDI/GSSG, TRXB1, DnaK mix, cofactor mix, Zn^2+^, GSSG, or 3C protease in the 96-well transfer plate. In the same order, the CFBs were added to ports A12–H12. To prepare the hemin stock, 13 mg hemin (Merck KGaA, Darmstadt, Germany, Cat. No. 51280) was dissolved in 10 mL of 0.1 M NaOH to yield a 2 mM solution, which was stored at –20 °C, protected from light. A 134 µM working solution for the DMC was then prepared by mixing 93.3 µL ddH_2_O with 6.7 µL of the 2 mM hemin stock.

Split GFP detector protein was added to ports A10, B10, G10, and H10. A wash buffer was added to C10 and D10 and an elution buffer to F10, H1, and H2. Blank buffers were added to H3 and H4. Universal control, complementation control, full workflow control, and expression controls were added to H5–H8, respectively.

Once DNA and reagents were dispensed, the streptactin beads supplied with the kit were washed three times using a magnetic bead separator and then resuspended to 30% (*v*/*v*). The E10 port was then loaded with 12 µL of the bead suspension. Degassed base fluid was connected to the eProtein Discovery™ instrument (Nuclera, Cambridge, UK) and injected through tube connectors to corner ports X2–X3 before running the automated program to initiate expression. The program was predefined to select the five (6 × 4 design) or ten (3 × 8 design) highest yielding SOL–CFB combinations per POI for down-stream purification. The eProtein Discovery^TM^ Cloud Software was used for data analysis and to generate expression and purification yield figures.

### 2.5. Scaled-Up Cell-Free Protein Synthesis and Purification

Scaled-up protein expressions were carried out in total volumes of 200 µL containing a PDI/GSSG CFB and 5 nM eGene constructs for either P17-AsPgb 1.5 or CUSF-BvPgb 1.2. Reaction mixtures were assembled on ice, briefly vortexed and centrifuged, then incubated at 29 °C for 15–18 h without agitation.

Following expression, proteins were purified using STREP tag affinity beads. Beads were washed and equilibrated in a wash buffer before the addition of the CFPS reaction mixture. Binding was performed for 30 min at room temperature with gentle agitation. Beads were then separated magnetically and washed three times in a wash buffer. The bound protein was eluted with 250 µL of an elution buffer. Crude reactions before and after CFPS, flow-through (FT), and eluted fractions were collected for the SDS-PAGE analysis.

The molar absorptivity at 280 nm (ε_280_) for P17-AsPgb 1.5 and CUSF-BvPgb 1.2 was calculated from their aa sequences using the ProtParam tool on the ExPASy server [47]. Protein concentrations were measured in triplicate by absorbance at 280 nm on a NanoDrop (Thermo Fisher Scientific, Waltham, MA, USA).

### 2.6. Mass Photometry

Microscope coverslips (24 × 50 mm, high precision) were cleaned with isopropanol and double-distilled water, then dried under a pressurized air stream. A silicone gasket with six wells was placed at the center of each coverslip to form measurement chambers. Filled wells contained a total volume of 20 µL with 20 nM protein concentration. An elution buffer from the scale-up was used as the negative control. Measurements were performed on a Refeyn TwoMP (Refeyn Ltd., Oxford, UK). One-minute videos were recorded using Refeyn AcquireMP 2024 R1 software, and data were analyzed with Refeyn DiscoverMP 2024 R1 software. Bovine serum albumin (66 kDa) and Immunoglobulin G (150 kDa and 300 kDa) proteins were used to generate the standard contrast-to-mass calibration curve.

### 2.7. Nano Differential Scanning Flourimetry

The melting temperatures (T_m_) of scaled-up CUSF-BvPgb 1.2 and P17-AsPgb 1.1 were determined using nanoDSF. Protein samples (10 µM in buffer G500) were loaded into standard nanoDSF-grade glass capillaries (10 µL; NanoTemper Technologies GmbH, Munich, Germany) and analyzed with a Prometheus NT.48 instrument (NanoTemper Technologies GmbH, Munich, Germany). Measurements were performed using a temperature gradient of 20–95 °C at a rate of 1.5 °C/min and an excitation power of 50%. Data were processed with PR.ThermControl v2.3.1 software (NanoTemper Technologies GmbH, Munich, Germany), and T_m_ was calculated from the first derivative of F330.

## 3. Results

### 3.1. Identification of Putative Phytoglobins in Avena sativa

Eight putative Pgbs were identified in the *Avena* reference genome [42]. The genetic location, transcript length, and orthogroup are seen in Table S1. The eight AsPgbs display high internal sequence similarity within each orthogroup (see Figure S1).

Expression profiling across seed, glume, spikelet, leaf, crown, and root tissues shows that AsPgbs are broadly expressed in oat as deposited in [42] (see Table 1). Transcripts for every AsPgb were detected in roots, with especially strong detection for AsPgb 1.4–1.6 and AsPgb 3.1–3.2. Crowns also expressed all Pgbs but at lower frequencies. Within class 1, the two orthogroups display contrasting tissue biases: AsPgb 1.1–1.3 is enriched in glume and leaf (17/21 and 21/33 detections, respectively) with minimal signal in seeds (5/72), whereas AsPgb 1.4–1.6 is largely seed- and root-biased (37/72 and 24/24) and absent from glume (0/21). By contrast, class 3 Pgbs (AsPgb 3.1/3.2) are detected across all tissues, with 100% detection in seed, glume, spikelet, leaf, and root, and 7/8 samples in crown.

### 3.2. Structural Comparison of Select Putative Class 1 AsPgbs to BvPgb 1.2

**Figure 1:**
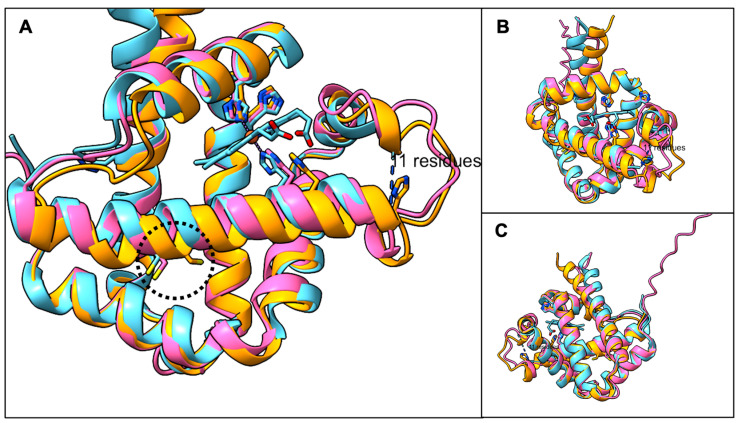
Backbone alignment of predicted structures of AsPgb 1.1 (orange) and AsPgb 1.5 (pink) against BvPgb 1.2 (blue). (**A**) Cysteine residues inscribed in dotted circle from left to right—C86, C84, C70—are similarly situated across the aligned Pgbs. (**B**) Heme proximal face. (**C**) Heme distal face, where the dimerization interface for BvPgb 1.2 sits [19].

Pairwise BLASTP comparisons of AsPgb 1.1 and 1.5 to BvPgb1.2 (171 aa) gave good alignments across most of the query (see Figures S2 and S3), with AsPgb 1.5 showing closer overall similarity. The capacity of globins to share low aa sequence identity, with some sharing as little as 10%, and still retain the structurally defining feature is well known [48]. A backbone chain pairing between the predicted structures of putative class 1 AsPgbs and the resolved structure of BvPgb 1.2 yielded sub-Ångström RMSD over pruned Cα pairs (see Table 2), suggesting a very good fit. Importantly, the position of the conserved cysteine residue appears very similar to that of BvPgb 1.2 (see Figure 1).

### 3.3. Construction of eGene Constructs

**Figure 2:**
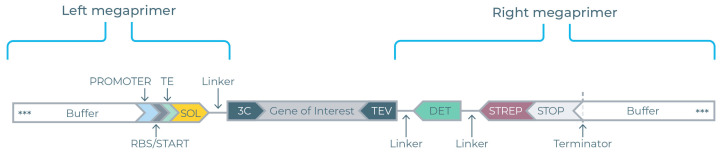
Description of the eGene construct. The gene of interest (GOI) insert is designed to complement the 3C protease binding site (3C) of the left megaprimer and the TEV protease binding site (TEV) of the right megaprimer. The left megaprimer contains the solubility tag (SOL) ahead of the translational regions containing a T7 promoter region, ribosome binding site (RBS)**,** and translation enhancer (TE) and is linked to the GOI insert at the 3C site. The right megaprimer is linked to the GOI insert at the TEV site and contains the split-GFP-based detection tag (DET), strep tag II (STREP), and terminator.

Linear DNAs encoding the BvPgb GOIs were inserted between two flanking megaprimers (see Figure 2) to create complete eGene constructs. Eight different left megaprimers were employed to generate the P17, CUSF, FH8, TRX, ZZ, SUMO, SNUT, and untagged eGenes. The right megaprimer includes the DET and STREP tags.

**Figure 3:**
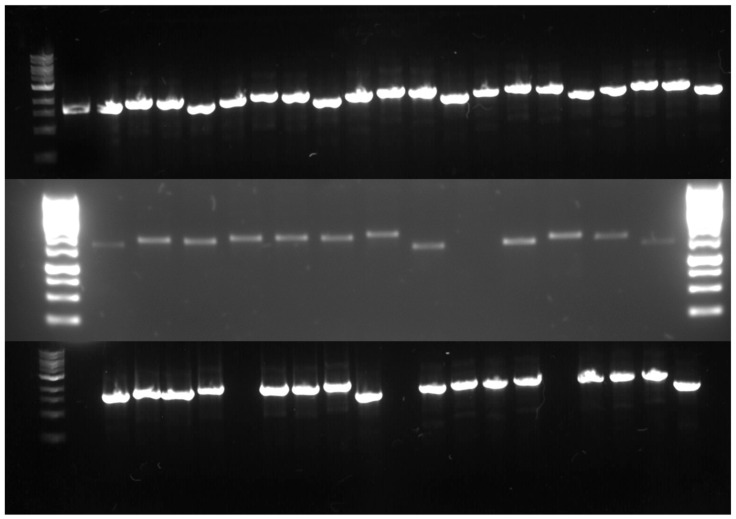
PCR products of the constructed eGenes™. Top, left to right: Ladder, control, AsPgb 1.1 with SOL tags P17, CUSF, FH8, and none, and the same for C70A, C84A, AsPgb 3.1, and C161A. Middle, left to right: Ladder, BvPgb 1.2 with SOL tags P17, CUSF, FH8, TRX, ZZ, SUMO, SNUT, none, and space; AsPgb 1.5 with SOL tags P17, CUSF, FH8, none, and ladder. Bottom: Ladder, C86A with SOL tags (from left to right) P17, CUSF, FH8, TRX, space, ZZ, SUMO, SNUT, none, and space, and the same for H-NOX.

The AsPgb GOIs were similarly constructed, foregoing the TRX, ZZ, SUMO, and SNUT primers. All eGene constructs were amplified through PCR, validated by gel electrophoresis (see Figure 3) and normalized before being loaded onto the DMCs.

### 3.4. CFPS Platform Run

**Figure 4:**
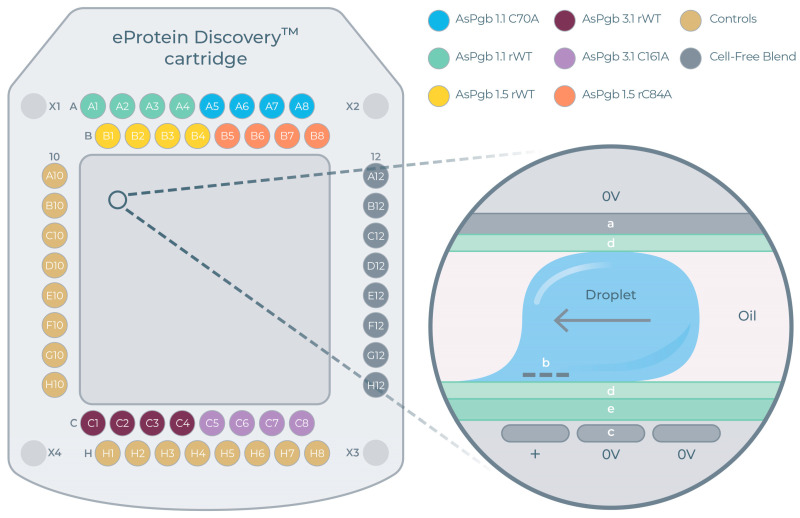
Digital microfluidics cartridge (DMC) for cell-free protein synthesis (CFPS). (**A**) DMC layout for AsPgb run. Ports A1–A8 for constructs AsPgb 1.1 and C70A, B1-B8 for AsPgb 1.5 and C84A, C1-C8 for AsPgb 3.1 and C161A, A10-H10 and H1-H8 for controls, and A12-H12 for cell-free blends (CFBs). Ports X2 and X3 are for the base fluid (oil). (**B**) Cross-section zoom-in with schematics of working principle of electrowetting used in DMC: (a) top electrode, (b) negative charge is induced at liquid/dielectric interface above (c) propulsion electrode receiving AC signal, (d) hydrophobic coating, and (e) dielectric film [49].

Each eGene construct was synthesized against eight different CFBs, yielding 8 × 8 = 64 and 4 × 8 = 32 unique expression conditions for each BvPgb and AsPgb construct, respectively. Reagents, controls, eGenes, and CFBs were loaded onto the DMC. The experimental layout for the AsPgb run can be seen in Figure 4A, with the mechanistic principle of electrowetting further explained in Figure 4B.

**Figure 5:**
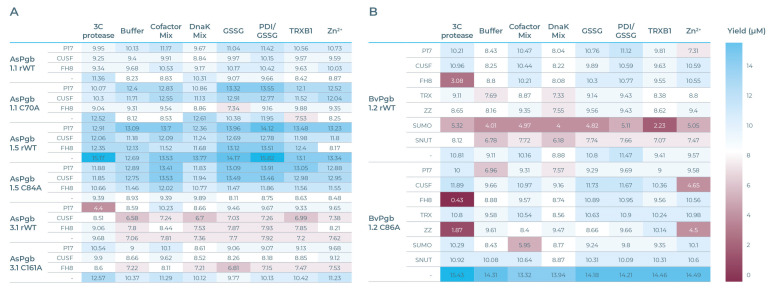
Heat maps of expression yields (µM), with blue and red cells having yields above or below 8 µM, respectively. (**A**) AsPgb heatmap, with SOL-tagged POI constructs to the left, in order P17, CUSF, FH8, and none (-). From top to bottom, AsPgb 1.1 rWT, C70A, AsPgb 1.5 rWT, C84A, AsPgb 3.1 rWT, and C161A. CFBs top, from left to right are ordered by 3C protease, buffer, cofactor mix, DnaK mix, glutathione disulfide (GSSG), GSSG mixed with protein disulfide isomerase (PDI/GSSG), thioredoxin reductase (TRXB1), and Zn^2+^. (**B**) BvPgb heatmap, with SOL-tagged POI constructs in order P17, CUSF, FH8, TRX, ZZ, SUMO, SNUT, and -. Topmost half are the BvPgb 1.2 rWT constructs and bottom half are the C86A constructs. CFBs are ordered the same as in (**A**). See Table S2 for detailed data.

The expression yields for the AsPgb run (see Figure 5A) and the BvPgb (see Figure 5B), measured through BiFC-induced fluorescence, show that a cysteine-to-alanine substitution can have significant impact on the workings of CFPS (see Table S2 for full data). For BvPgb 1.2, the SUMO tag suppresses rWT expression but has negligible impact on the C86A variants. AsPgb 1.5 sans SOL tag gave the highest yielding expression, whereas its C84A counterpart had the lowest yielding expression across all CFBs. Perhaps most striking was the difference the 3C protease-supplemented CFB had on AsPgb 3.1 and its C161A variant. CUSF markedly enhanced yields for alanine-substituted class 1 AsPgb variants. Generally, the most beneficial additives were GSSG and PDI/GSSG.

**Figure 6:**
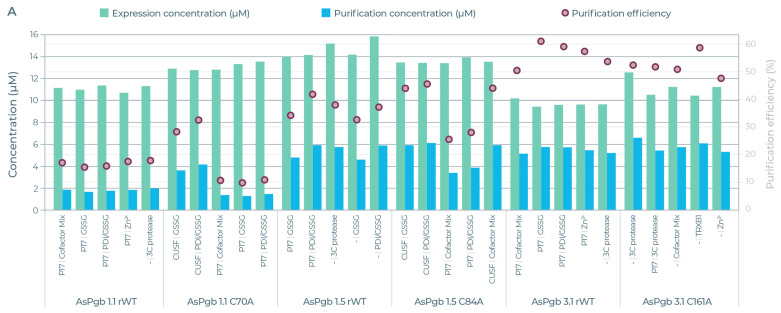

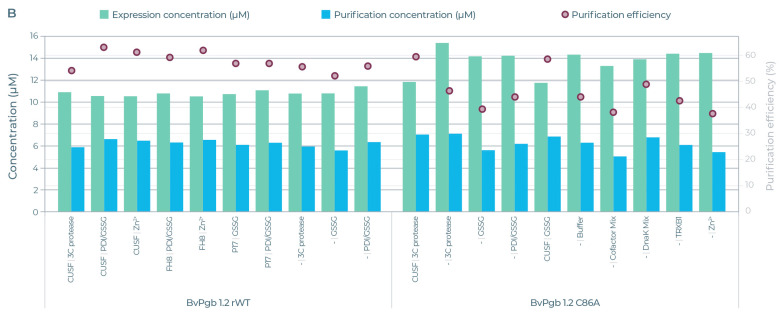
Expression yields (green) and purified yield (blue) against left axis (µM) and efficiency (red circle) against right axis (%) for selected AsPgb and BvPgb constructs. Each selected AsPgb POI had 5 different compositions, and each selected BvPgb POI had 10 different compositions. (**A**) From left to right: AsPgb 1.1 rWT, C70A, AsPgb 1.5 rWT, C84A, AsPgb 3.1 rWT, and C161A. (**B**) Left BvPgb 1.2 rWT, right C86A. See Table S2 for detailed data.

The five AsPgb and ten BvPgb compositions with the highest yields (see Table S2 for full data) were selected for downstream strep-based purification (see Figure 6). Here too, alanine substitution appeared to have a big effect. For example, the lower purification efficiency of the C86A variant was compensated by its high expression yields, giving a comparative yield to that of its parental BvPgb rWT (see Figure 6B). Conversely, the high purification efficiency for class 3 AsPgbs outweighed their low expression when compared to the other AsPgbs (see Figure 6A).

The full DMC run for the 3 × 8 and 6 × 4 design can be seen in Video S1 and Video S2, respectively. Purified expression yields spanned 5.06–7.14 µM for C86A and 5.61–6.69 µM for the BvPgb 1.2 rWT. For the Avena Pgbs, purified expression yields spanned 1.73–2.04, 4.66–5.93, and 5.2–5.79 µM for AsPgb 1.1, 1.5, and 3.1, respectively. For C70A, C84A, and C161A, the purified yields spanned 1.35–4.19, 3.45–6.16, and 5.37–6.62 µM, respectively.

### 3.5. Scaled-Up In Vitro Protein Production and Assessment of Protein Oligomeric State and Stability

Since AsPgb 1.5 shared the highest similarity with BvPgb 1.2, it was selected for the 200 µL scale-up of CFPS alongside BvPgb 1.2. The constructs differed in optimal SOL tags, and P17 was opted for AsPgb 1.5 and CUSF was opted for BvPgb 1.2. The PDI/GSSG CFBs were employed due to their overall positive performance in the DMC runs. The scaled-up purified yield for AsPgb was 22 µM (0.69 mg/mL) and 20 µM (0.77 mg/mL) for BvPgb.

**Figure 7:**
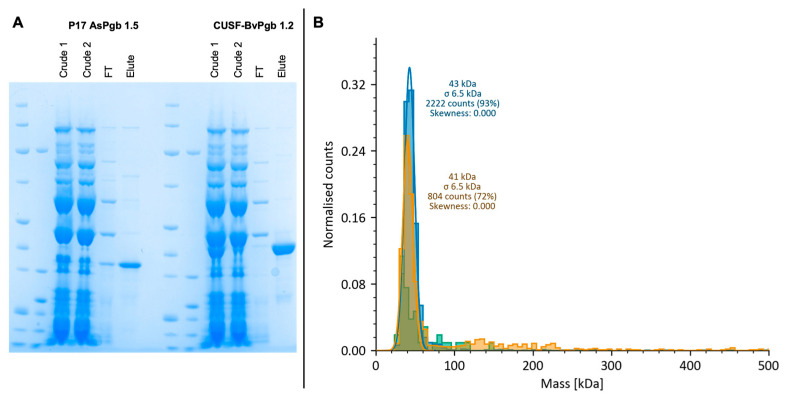
Assessment of scale-up and oligomeric state of P17-AsPgb 1.5 (31 kDa) and CUSF-BvPgb 1.2 (38 kDa). (**A**) SDS-PAGE of AsPgb scale-up (left) and BvPgb scale-up (right) shows successful isolation of proteins of expected size in eluted row. (**B**) Mass photometry (MP) of P17-AsPgb 1.5 (yellow), CUSF-BvPgb 1.2 (blue), and Nuclera buffer (green) reveals single peaks at comparative masses for the monomeric form of the POIs.

The different steps of the scale-up were assessed through SDS-PAGE, with eluted protein bands having molecular weights corresponding to the expected POI size (i.e., of 31 and 38 kDa) as seen in Figure 7A.

MP measurements of BvPgb had 93% of counts at 43 ± 6.5 kDa, whereas AsPgb had 72% of counts at 41 ± 6.5 kDa, suggesting that both POIs were obtained in a monomeric state (see Figure 7B). The higher-than-expected mass as reported by MP can be explained by known instrumental upward biases for molecules near the lower detection limit of 30 kDa [50,51].

The thermal stability measurements were intended to provide preliminary insight into relative stability trends rather than a full thermodynamic characterization. The POIs were assessed using nano DSF (see Figure S4). First derivatives from counts.mm at 350 nm (see Figure S4A) and 330 nm (see Figure S4B) and the ratio (see Figure S4C) indicate a T_m_ of 55 °C for BvPgb, with results inconclusive for the AsPgb 1.2. Scattering results suggest that the BvPgb experiences unfolding-induced aggregation at >70 °C (see Figure S4D).

## 4. Discussion

Protein science depends on having enough protein to research. To this there are two approaches: “make more” or “make do”. Considerable advances in lowering sample consumption have been made through miniaturization, automation [52], and novel adaptations [25]. This has lowered the entry barrier for fingerprinting proteins [52], and demand for rapid protein prototyping is a natural outcome. Traditional recombinant expression protocols can take weeks, or even months, to develop. Screening multiple parameters in parallel can accelerate or aid in trouble-shooting such developments [53]. The CFPS platform used here provided results on a total of 192 unique expression conditions, with 30 downselected for purification, within 48 h. The benchmarking screen readily highlighted, for example, that SUMO-tagging BvPgbs consistently hindered expression and is therefore best avoided (see Figure 5B). AsPgb 3.1 can similarly be recognized as a potentially challenging protein to express (see Figure 5A).

Representative members (AsPgb 1.1, 1.5, 3.1) were chosen to capture each orthogroup, since remaining AsPgbs within a group share >90% sequence identity (see Figure S1) and similar predicted structures, rendering them suitable proxies for expression behavior. Future work may expand screening to additional homologs for finer comparative analysis.

Since key components of the *E. coli* translational machinery are retained, CFPS can to some extent model the expression conditions experienced in vivo [54]. Results from DMC runs can therefore provide valuable insight into the biological requirements of POIs in addition to guiding the development of suitable expression protocols.

All SOL tags were positioned N-terminally to ensure uniform construct design and compatibility with the current DMC workflow. Nevertheless, some proteins may fold more efficiently with C-terminal or internal fusions, as tag position can influence co-translational folding and accessibility [55]. Each SOL tag was screened for distinct properties known to enhance protein expression and folding. The P17 tag, a small (3.8 kDa) hydrophilic fragment derived from the tail protein of bacteriophage T7, has been reported to improve solubility and thermostability [34]. FH8, a 7.5 kDa thermostable antigen from *Fasciola hepatica*, is unusual in that it can both increase solubility and serve as a robust purification handle [36]. The CUSF tag (9.9 kDa), a periplasmic beta-barrel protein from the CusCBFA efflux complex, was included for its compact, stable fold; its ability to improve soluble yields; and its natural role in increasing metallo-toxic resistance in *E. coli* [35]. The HSUMO3 (SUMO) tag (11.5 kDa), a human small ubiquitin-like modifier, acts almost as a detergent-like solubilizer and has been widely applied to recover proteins otherwise prone to aggregation [39]. Similarly, the TRX tag (11.7 kDa thioredoxin from *E. coli*) is a classical solubility enhancer that promotes correct folding [37]. Larger tags were also tested, including SNUT (16.7 kDa), derived from *Staphylococcus aureus* sortase A, which has been developed as a “ubiquitous” solubility enhancer across diverse protein classes [40]. In addition, the ZZ domain (13.2 kDa), a derivative of protein A from *S. aureus*, was incorporated as it provides both solubility benefits and potential utility in IgG-binding applications [38]. Interestingly, successful SOL tags were all smaller than 10 kDa, in contrast to SNUT (16.7 kDa), TRX (11.7 kDa), ZZ (13.2 kDa), and SUMO (11.5 kDa) [34,35,36,37,38,39,40].

For the BvPgb DMC benchmarking, we saw that the C86A variant benefited more by excluding SOL tags than the rWT, which instead benefitted most from the P17, FH8, and CUSF tags. The ten highest yielding expression conditions for each BvPgb POI were selected for purification. Purified yields spanned 5.06–7.14 µM for C86A and 5.61–6.69 µM for rWT. The alanine-substituted AsPgbs also outperformed their parental molecules with purified yield spans of 1.35–4.19 µM for C70A vs. 1.73–2.04 µM for AsPgb 1.1, 3.45–6.16 µM for C84A vs. 4.66–5.93 µM for AsPgb 1.5, and 5.37–6.62 µM for C161A vs. 5.2–5.79 µM for AsPgb 3.1. Alanine substitutions were selected to probe the structural impact of cysteine removal, since this residue is involved in oxidative stability of the protein [19]. However, it could be valuable to explore alternative substitutions, such as serine, to disentangle side-chain-specific inactive residues like alanine versus possible hydrogen binding effects associated with serine on Pgb expression and stability. Although the class 1 AsPgbs share close structural alignment with BvPgb 1.2, their divergent expression outcomes can also stem from subtle sequence-dependent factors such as local hydrophobicity or surface charge [8] rather than just gross structural differences. Future computational studies, including electrostatic surface mapping and stability simulations, could further elucidate how these features modulate CFPS expression efficiency.

Each CFB had at least one instance of downstream purification. The control buffer only accounted for 2% of selected CFBs, suggesting that Pgb expression can largely be improved by specific additive compositions. GSSG (22%) along with the PDI/GSSG (26%) accounted for 48% of CFB conditions selected for downstream purification. The PDI addition was not only a boon to rWT POIs, suggesting that potential disulfide bond formation is not the significant determinant here. Overall, the GSSG and PDI/GSSG dominance suggests that redox control on oxidizing environments is the main factor that behooves Pgb expression. This effect is consistent with redox regulation observed in other heme and globin systems and highlights how tuning the oxidative balance can improve recombinant expression of redox-sensitive proteins [8]. It is therefore surprising that TRXB1 (4%) had such a low representation, since it specifically protects proteins from oxidative aggregation [56]. The DnaK mix also suppresses aggregation and was not found to perform well (2%). The cofactor mix (14%) and Zn^2+^ (12%) were in fact more beneficial than these chaperones; suggesting that added cofactors can play useful auxiliary roles in Pgb expression.

Since 19% of the 3C protease CFBs were selected for purification, it is likely that the SOL tags can intrinsically impede Pgb expression. It also proves that SOL tags are non-essential for the solubility of these Pgbs. This was further supported by the fact that POIs without an SOL tag accounted for 40% of the SOL tag conditions selected for downstream purification, comparable to P17 at 36%, CUSF at 20%, and FH8 at 4%. Coincidentally, our recombinant protocols do not employ SOL-tagged Pgb constructs.

The scale-up of P17-AsPgb 1.5 yielded 22 µM, or 0.69 mg/mL, similar to the CUSF-BvPgb 1.2 scale-up yield of 20 µM, or 0.77 mg/mL BvPgb. A limited number of scale-up reactions were chosen to provide representative validation rather than exhaustive coverage of expression patterns. Additional studies will extend scale-up to additional constructs to further assess the platform’s robustness and scalability across diverse protein classes. MP measurements revealed that the proteins were expressed in monomeric form, with nanoDSF measuring T_m_ of 55 °C for the BvPgb. The results for the AsPgb 1.5 were inconclusive, and observed peaks are more likely to reflect signal background than actual transitions (see Figure S4). It is likewise interesting to see that the MP result for P17-AsPgb shows only 72% of the protein (see Figure 7). BvPgb 1.2, as expressed by our recombinant protocols, has three distinct transition events, as measured by nanoDSF, at 53 °C, 67 °C, and 86 °C. The two lower transition events are absent when BvPgb 1.2 is in a cyanide-bound form [45]. However, it should also be noted that previously measured BvPgb 1.2 was dimeric, which is likely to confer thermal stability to the protein [57]. The thermal stability assessment aimed to provide preliminary insight rather than full thermodynamic characterization. Although only BvPgb 1.2 yielded a clear T_m_, its higher stability aligns with earlier reports linking dimerization to thermal resistance. Upcoming studies employing differential scanning calorimetry or structural dynamics simulations may reveal possible conformational transitions underlying these stability differences.

It has been shown that heme incorporation is essential for both structural integrity and oligomerization of globins [58,59,60]. Taken together, the results suggest that these Pgbs were expressed in a soluble apo-form. Achieving soluble apo-form Pgbs from the CFPS platform is a non-trivial achievement since apo-form globins tend to self-associate and aggregate [61,62]. This can in part explain the relatively low number of deposited apo-form globin structures in the PDB [63].

Altogether, the data show that CFPS platforms can delineate protein-specific requirements for Pgbs with a resolution difficult to achieve in recombinant systems. The clear dependence on redox additives, coupled with the variable effects of solubility tags, indicates that expression bottlenecks are multifactorial and context-dependent. Incorporating targeted cofactors or chaperones that facilitate heme insertion will likely be required to achieve fully functional holo-Pgbs. Glyceraldehyde-3-phosphate dehydrogenase (GAPDH) has recently emerged as a moonlighter involved in heme trafficking [64] and heme maturation of hemoglobin (Hb) and myoglobin (Mb) [65], suggesting that it may also play a role in heme insertion in Pgbs. Supporting this, we have recently shown that BvPgb 1.2 binds strongly to GAPDH in vitro [66].

Such refinements would position CFPS not only as a screening tool but also as a bona fide discovery platform for elucidating the minimal set of cellular factors required for POI biogenesis, extending beyond heme proteins. Indeed, the stripped-down nature of CFPS systems makes it possible to link the restoration of expression or folding directly to the addition of a missing cofactor or chaperone. Likewise, the platform can also highlight how a single aa substitution can alter what constitutes a favorable expression condition.

In contrast to earlier digital microfluidic CFPS systems such as Liu et al. [67], which required external control and manual reagent preparation, the Nuclera platform integrates fully automated droplet manipulation, reagent mixing, on-chip purification, and real-time fluorescence quantification.

Future studies should integrate heme maturation factors and ligand-binding analyses to confirm holo-protein formation and activity, extending the current CFPS-based screening toward functional characterization of Pgbs. This could be performed using UV–Vis spectroscopy. In this study, yield and solubility served as initial indicators of successful expression suitable for high-throughput screening. Nonetheless, we recognize that additional quality parameters (e.g., monodispersity, pH- or ion-dependent stability, and aggregation behavior) would provide valuable functional context. Subsequent work will aim to integrate these evaluations to better connect CFPS screening outcomes with downstream biochemical performance.

Although the CFPS platform isolates translational effects from endogenous regulation, the observed tissue-specific expression of Pgbs may still reflect intrinsic sequence adaptations influencing folding or stability. Investigating whether naturally low-abundance Pgbs also exhibit reduced CFPS expression could reveal connections between in vivo regulation and in vitro translatability. Such comparative analyses represent an exciting direction for upcoming studies bridging biological context and CFPS screening behavior.

## 5. Conclusions

This study demonstrates how a CFPS combined with digital microfluidics provides a powerful platform for rapidly screening expression conditions of challenging heme proteins such as Pgbs. By benchmarking BvPgb 1.2 and AsPgbs alongside cysteine-to-alanine variants, we revealed how single aa substitutions, CFB and SOL tag choices, can dramatically alter expression and purification outcomes. The consistent improvement observed with redox-modifying additives underscores the central role of oxidative environments in Pgb stability, while the lack of heme incorporation highlights a potential need for additional cofactors or chaperones to achieve functional holo-proteins. Beyond accelerating protocol development, these findings provide mechanistic insights into the determinants of Pgb expression, suggesting that CFPS can be leveraged not only for production but also for dissecting protein-specific biogenesis requirements. The findings should be interpreted with the recognition that (i) heme content was not directly verified, (ii) the number of replicates and constructs tested was limited, and (iii) no functional assays were performed. These aspects represent current limitations of the study and will be addressed in future work focusing on heme incorporation and activity validation. The reason is that we wish to integrate targeted maturation factors such as GAPDH to support heme insertion and extend this strategy to other intractable globins, which is beyond the present study.

## Figures and Tables

**Table 1:** Putative phytoglobins (Pgb) from *Avena sativa* (AsPgbs) grouped by orthogroup and their detection across selected tissues [42]. Values are counts of samples with a detection; *n* indicates the number of samples per tissue. Orthogroup totals are summed across members, with denominators showing the maximum possible detections.

		Tissue					
Orthogroup	Species ID	Seed *n* = 24	Glume *n* = 7	Spikelet *n* = 8	Leaf *n* = 11	Crown *n* = 4	Roots *n* = 8
OG0017468	AsPgb 1.1	0	4	0	4	1	3
	AsPgb 1.2	5	6	2	7	3	8
	AsPgb 1.3	0	7	1	10	3	8
	**Total** **:**	5/72	17/21	3/24	21/33	7/12	19/24
OG0007273	AsPgb 1.4	5	0	1	0	1	8
	AsPgb 1.5	16	0	2	1	1	8
	AsPgb 1.6	16	0	3	0	1	8
	**Total:**	37/72	0/21	6/24	1/33	3/12	24/24
OG0012302	AsPgb 3.1	24	7	8	11	4	8
	AsPgb 3.2	24	7	8	11	3	8
	**Total:**	48/48	14/14	16/16	22/22	7/8	16/16

**Table 2:** Alignment of predicted structures of putative AsPgbs towards reference structure of class 1 Pgb from *Beta vulgaris* (BvPgb 1.2, PDB ID: 7ZOS), with score, pruned atom pairs, and root-mean-square difference (RMSD) in Å.

Protein	Alignment Score (A.U)	Pruned Atom Pairs (#)	RMSD (Å)
AsPgb 1.1	534.5	101	0.859
AsPgb 1.5	603.7	130	0.945

## Data Availability

The original contributions presented in this study are included in the article/Supplementary Material. Further inquiries can be directed to the corresponding author.

## Supplementary Materials

The following supporting information can be downloaded at: https://www.mdpi.com/article/10.3390/antiox14111317/s1, Figure S1: Multiple sequence alignment of AsPgbs by orthogroup; Figure S2: BLASTp of AsPgb 1.1 queried against BvPgb 1.2; Figure S3: BLASTp of AsPgb 1.5 queried against BvPgb 1.2; Figure S4: Thermal stability of P17-AsPgb 1.5 and CUSF-BvPgb 1.2; Table S1: Putative *Avena sativa* phytoglobins (AsPgbs) grouped by orthogroup, with chromosome positions, strand direction, and translated protein size by number of amino acids (aa); Table S2: CFPS expression and purification yields from DMC runs; Video S1: DMC 3 × 8 layout run; Video S2: DMC 6 × 4 layout run. Timestamped descriptions for both Video S1 and Video S2: Loaded reagent reservoirs (00:00) are first split and dispensed as smaller droplets (00:07). Droplets are arranged into arrays (00:09), before CFBs and normalized eGene droplets are merged and mixed (00:10). The mixed droplets are incubated for CFPS and are subsequently split into two droplets (00:12). In parallel, bead mixing occurs (left side on DMC). The detection probe is merged and mixed with the expressed POI (00:13) and allowed to incubate for bimolecular fluorescence complementation (BiFC) before yield quantification. The detector-complemented expressed proteins are discarded, and the remaining uncomplemented droplets are filtered for purification based on expression yield (00:19). The selected constructs are merged with bead droplets for purification (00:20), washed twice (00:24, 00:26), and eluted (00:30). The eluted POIs are mixed with the detection probes (00:32) to measure purified yield.

## Abbreviations

The following abbreviations are used in this manuscript:

aa	Amino acid
AsPgb	*Avena sativa* phytoglobin
BvPgb	*Beta vulgaris* phytoglobin
BiFC	Bimolecular fluorescence complementation
C70A, C84A, C86A, C161A	Cysteine-to-alanine substitutions at residues 70, 84, 86, and 161 for AsPgb 1.1, AsPgb 1.5, BvPgb 1.2, and AsPgb 3.1, respectively
CFB	Cell-free blend
CFCR	Cell-free core reagent
CFPS	Cell-free protein synthesis
Cofactor mix	CFB additive containing NAD, acetyl-CoA, FAD, SAM, and PLP
CUSF	Solubility tag derived from CusCBFA efflux complex (periplasmic β-barrel protein)
DMC	Digital microfluidics cartridge
NanoDSF	Nano differential scanning fluorimetry
eGene™	Linear DNA construct for CFPS expression
FH8	Solubility tag derived from *Fasciola hepatica* antigen
GAPDH	Glyceraldehyde-3-phosphate dehydrogenase
GFP11	Split GFP probe (11th β-strand)
GOI	Gene of interest
GSSG	Glutathione disulfide
H-NOX	Heme–nitric oxide/oxygen-binding protein
K_D_	Dissociation constant
MP	Mass photometry
NAD	Nicotinamide adenine dinucleotide
P17	Solubility tag derived from bacteriophage T7 tail protein
PDI	Protein disulfide isomerase
PLP	Pyridoxal phosphate
POI	Protein of interest
rWT	Recombinant wild-type
SAM	S-adenosylmethionine
SNUT	Solubility tag derived from *Staphylococcus aureus* sortase A
SOL tag	Solubility tag (general term)
STREP	Strep tag II
SUMO	Small ubiquitin-like modifier (HSUMO3 tag)
T_m_	Melting temperature
TRX	Thioredoxin (solubility tag)
TRXB1	Thioredoxin reductase
ZZ	Solubility tag derived from protein A (*Staphylococcus aureus*)
Zn^2+^	Zinc chloride additive
