# Supplementary material for: Digital Microfluidics-Driven Cell-Free Protein Synthesis Platform Reveals Expression and Stability Determinants for Phytoglobins and Cysteine-to-Alanine Substituted Variants
Source: Antioxidants (Basel). 2025 Oct 31;14(11):1317. doi: 10.3390/antiox14111317 (PMC12649544; doi:10.3390/antiox14111317)
Supplement: Supplementary file 1 [file antioxidants-14-01317-s001.zip › Supplementary Information.pdf]

Supplementary Information

```
Orthogroup: OG0017468

AsPgb1.2        MGFSEAQEELVLRSWKAMKPDSESIALKFFLRIFEIAPAAKPMFPFLREAGEDAPLESHP
AsPgb1.3        MGFSEAQEELVLRSWKAMKPDSESIALKFFLRIFEIAPAAKAMFPFLREAGEDAPLESHP
AsPgb1.1        MGFSEAQEELVLRSWKAMKPDSESIALNFFLRIFEIAPAAKPMFPFLRED---APLESHP
                ***************************:*************.*******    *******

AsPgb1.2        KLKAHAVTVFVMACESATQLRKTGDVKVREATLRRLGATHVKAGVADAHFEVVKTALLDT
AsPgb1.3        KLKAHAVTVFVMACESATQLRKTGDVKVREATLRRLGATHVKAGVADAHFEVVKTALLDT
AsPgb1.1        KLRAHAVTVFVMACESATQLRKTGDVKVREATLRRLGATHVKAGVADAHFEVVKTALLDT
                **:*********************************************************

AsPgb1.2        IEGAVPEMWTPEMKGAWEEAYDQLAAAIKEEMKIAASASA
AsPgb1.3        IERAVPEMWTPEMKGAWEEAYDQLAAAIKEEMKIAASASA
AsPgb1.1        IEGAVTEMWTPEMKGAWEEAYDQLAAAIKEEMKIAASASA
                ** **.**********************************

Orthogroup: OG0007273

AsPgb1.4        MSAVEGNSAAG--GAVVFSEEQEALVLKSWAIMKKDSANLGLRFFLKIFEIAPSAKQMFP
AsPgb1.5        MSAVEGSSASG--AAVVFSEEQEALVLKSWAIMKKDSANLGLRFFLKIFEIAPSAKQMFP
AsPgb1.6        MSAVEGNIASGGGGAVVFSEEQEALVLKSWAIMKKDSANLGLRFFLKIFEIAPSAKQMFP
                ******. *:*  .**********************************************

AsPgb1.4        FLRNSDVPLETNPKLKTHAVSVFVMTCEAAAQLRKAGKITVRETSLKRLGGTHVKYGVAD
AsPgb1.5        FLRDSDVPLETNPKLKTHAVSVFVMTCEAAAQLRKAGKITVRETTLKRLGGTHVKYGVAD
AsPgb1.6        FLRNSDVPLETNPKLKTHAVSVFVMTCEAAAQLRKAGKITVRETTLKRLGGTHLKYGVAD
                ***:****************************************:********:******

AsPgb1.4        GHFEVTRFALLDTIKEAVPADMWGPEMKNAWGEAYDQLVAAIKQEMKPSA
AsPgb1.5        GHFEVTRFALLDTIKGAVPADMWGPEMKTAWGEAYDQLVAAIKQEMKPSA
AsPgb1.6        GHFEVTRFALLETIKEAVPADMWGPEMRNAWGEAYDQLVAAIKQEMKPSA
                ***********:*** ***********:.*********************

Orthogroup: OG0012302

AsPgb3.1        MQSLQDKASEWSGVAAADAFAIDEVNVFEALGGTPQPFVDLSTNFYTRVYEDEEEWFREI
AsPgb3.2        MQSLQDKASEWSGVAAADAFAIDEVNVFEALGGTPQPFVDLSTNFYTRVYEDEEEWFREI
                ************************************************************

AsPgb3.1        FSGSKKEDAIQNQYEFLVQRMGGPPLFSQRRGHPALIGRHRPFPVTHQAAERWLHHMQQA
AsPgb3.2        FSESKKEDAIQNQYEFLVQRMGGPPLFSQRRGHPALIGRHRPFPVTHRAAERWLHHMQQA
                ** ********************************************:************

AsPgb3.1        LDSTESINSDTKTKMMYFFRHTAYFLVAGNEMTRQAQVVPPCKHATSKPAE
AsPgb3.2        LDSTESINSDTKTKMMYFFRHTAYFLVAGNEMTRQAQVVPPCKHAASKPAE
                *********************************************:*****
```

Figure S1: Multiple sequence alignment of AsPgbs by orthogroup

```
Query: BvPgb1.2 Query ID: lcl|Query_5197662 Length: 171


>AsPgb1.1
Sequence ID: Query_5197664 Length: 156
Range 1: 2 to 149


Score:179 bits(455), Expect:3e-63,
Method:Compositional matrix adjust.,
Identities:84/149(56%), Positives:110/149(73%), Gaps:1/149(0%)


Query  17    FTEEQEALVVQSWNVMKKNSAELGLKLFLKIFEIAPTAKKMFSFVRDSDVPLEQNQKLKG  76
             F+E QE LV++SW  MK +S  + L  FL+IFEIAP AK MF F+R+ D PLE  + KL+
Sbjct  2     FSEAQEELVLRSWKAMKPDSESIALNFFLRIFEIAPAAKPMFPFLRE-DAPLESHPKLRA  60


Query  77    HAMSVFVMTCKSAAQLRKAGKVTFGESSLKHMGSVHLKYGVVDEHFEVTRFALLETIKEA  136
             HA++VFVM C+SA QLRK G V   E++L+ +G+ H+K GV D HFEV + ALL+TI+ A
Sbjct  61    HAVTVFVMACESATQLRKTGDVKVREATLRRLGATHVKAGVADAHFEVVKTALLDTIEGA  120


Query  137   VPEMWSPEMKNAWAEAFNHLVAAIKAEMQ  165
             V EMW+PEMK AW EA++ L AAIK EM+
Sbjct  121   VTEMWTPEMKGAWEEAYDQLAAAIKEEMK  149
```

Figure S2: BLASTp of AsPgb 1.1 queried against BvPgb 1.2

```
Query: BvPgb1.2 Query ID: lcl|Query_5197662 Length: 171

>AsPgb1.5
Sequence ID: Query_5197665 Length: 167
Range 1: 8 to 164

Score:234 bits(598), Expect:8e-85,
Method:Compositional matrix adjust.,
Identities:110/157(70%), Positives:133/157(84%), Gaps:1/157(0%)

Query  10   ASDGTVIFTEEQEALVVQSWNVMKKNSAELGLKLFLKIFEIAPTAKKMFSFVRDSDVPLE  69
            AS   V+F+EEQEALV++SW +MKK+SA LGL+ FLKIFEIAP+AK+MF F+RDSDVPLE
Sbjct  8    ASGAAVVFSEEQEALVLKSWAIMKKDSANLGLRFFLKIFEIAPSAKQMFPFLRDSDVPLE  67

Query  70   QNQKLKGHAMSVFVMTCKSAAQLRKAGKVTFGESSLKHMGSVHLKYGVVDEHFEVTRFAL  129
             N KLK HA+SVFVMTC++AAQLRKAGK+T  E++LK +G  H+KYGV D HFEVTRFAL
Sbjct  68   TNPKLKTHAVSVFVMTCEAAAQLRKAGKITVRETTLKRLGGTHVKYGVADGHFEVTRFAL  127

Query  130  LETIKEAVP-EMWSPEMKNAWAEAFNHLVAAIKAEMQ  165
            L+TIK AVP +MW PEMK AW EA++ LVAAIK EM+
Sbjct  128  LDTIKGAVPADMWGPEMKTAWGEAYDQLVAAIKQEMK  164
```

Figure S3: BLASTp of AsPgb 1.5 queried against BvPgb 1.2

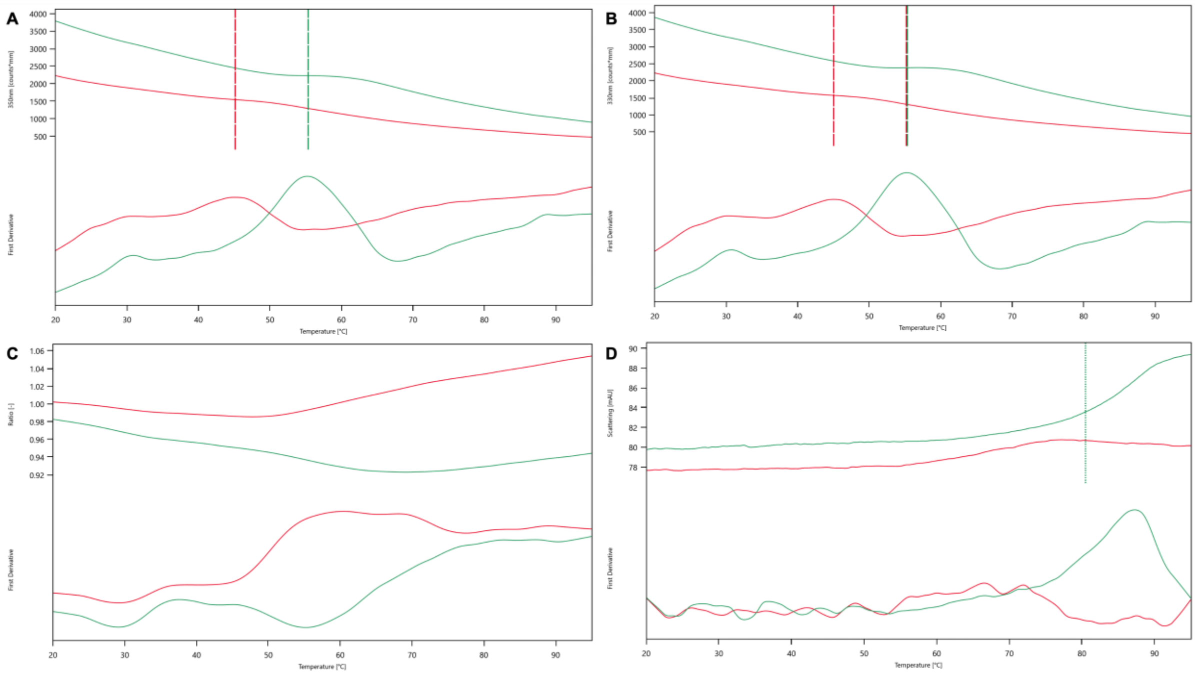

Figure S4. Thermal stability of P17-AsPgb 1.5 (red) and CUSF-BvPgb 1.2 (green) as determined by nano differential scanning fluorimetry (nano-DSF) over temperature span from 20–95 °C. (A) Thermogram at 350 nm, (B) thermogram at 330 nm (C) ratio of fluorescence (350/330), (D) scattering plot (mAU). All subplots include first derivative underneath.

Table S1: Putative *Avena sativa* phytoglobins (AsPgbs) grouped by orthogroup, with chromosome positions, strand direction, and translated protein size by number of amino acids (aa). Genetic location, transcript length, protein size and orthogroups of identified putative AsPgbs

Orthogroup	Species ID	Chromosome	Start	End	Direction	Protein size
OG0017468	AsPgb 1.1	1D	334876319	334877337	-	157 aa
	AsPgb 1.2	1A	353004257	353004822	-	160 aa
	AsPgb 1.3	1A	492042087	492042674	-	160 aa
OG0007273	AsPgb 1.4	1D	423036905	423038805	+	168 aa
	AsPgb 1.5	5A	15179003	15180934	-	168 aa
	AsPgb 1.6	4C	651979152	651981224	+	170 aa
OG0012302	AsPgb 3.1	7D	400521885	400525600	-	171 aa
	AsPgb 3.2	2A	405584564	405588208	-	171 aa

Table S2: CFPS expression and purification yields from DMC runs

	Protein of Interest						Expression yields		Purified yields
Construct	Species	##	Var	SOL	CFB	MW (kDa)	(mg/mL)	(uM)	(uM)
_-AsPgb1.1-rWT	AsPgb	1.1	rWT	_	3C protease	23.41	0.27	11.36	2.04
P17-AsPgb1.1-rWT	AsPgb	1.1	rWT	P17	Cofactor Mix	30.01	0.34	11.17	1.94
P17-AsPgb1.1-rWT	AsPgb	1.1	rWT	P17	Zn2+	30.01	0.32	10.73	1.9
P17-AsPgb1.1-rWT	AsPgb	1.1	rWT	P17	PDI/GSSG	30.01	0.34	11.42	1.84
P17-AsPgb1.1-rWT	AsPgb	1.1	rWT	P17	GSSG	30.01	0.33	11.04	1.73
P17-AsPgb1.1-rWT	AsPgb	1.1	rWT	P17	TRXB1	30.01	0.32	10.56	0
FH8-AsPgb1.1-rWT	AsPgb	1.1	rWT	FH8	Cofactor Mix	33.73	0.36	10.53	0
FH8-AsPgb1.1-rWT	AsPgb	1.1	rWT	FH8	PDI/GSSG	33.73	0.35	10.42	0
_-AsPgb1.1-rWT	AsPgb	1.1	rWT	_	DnaK Mix	25.05	0.26	10.31	0
FH8-AsPgb1.1-rWT	AsPgb	1.1	rWT	FH8	GSSG	33.73	0.34	10.17	0
CUSF-AsPgb1.1-rWT	AsPgb	1.1	rWT	CUSF	PDI/GSSG	36.12	0.37	10.15	0
P17-AsPgb1.1-rWT	AsPgb	1.1	rWT	P17	Buffer	30.01	0.3	10.13	0
FH8-AsPgb1.1-rWT	AsPgb	1.1	rWT	FH8	Zn2+	33.73	0.34	10.03	0
CUSF-AsPgb1.1-rWT	AsPgb	1.1	rWT	CUSF	GSSG	36.12	0.36	9.97	0
P17-AsPgb1.1-rWT	AsPgb	1.1	rWT	P17	3C protease	23.41	0.23	9.95	0
CUSF-AsPgb1.1-rWT	AsPgb	1.1	rWT	CUSF	Cofactor Mix	36.12	0.36	9.91	0
FH8-AsPgb1.1-rWT	AsPgb	1.1	rWT	FH8	Buffer	33.73	0.33	9.68	0
P17-AsPgb1.1-rWT	AsPgb	1.1	rWT	P17	DnaK Mix	30.01	0.29	9.67	0
_-AsPgb1.1-rWT	AsPgb	1.1	rWT	_	PDI/GSSG	25.05	0.24	9.66	0
FH8-AsPgb1.1-rWT	AsPgb	1.1	rWT	FH8	TRXB1	33.73	0.32	9.63	0
CUSF-AsPgb1.1-rWT	AsPgb	1.1	rWT	CUSF	Zn2+	36.12	0.35	9.59	0
CUSF-AsPgb1.1-rWT	AsPgb	1.1	rWT	CUSF	TRXB1	36.12	0.35	9.57	0
CUSF-AsPgb1.1-rWT	AsPgb	1.1	rWT	CUSF	Buffer	36.12	0.34	9.4	0
FH8-AsPgb1.1-rWT	AsPgb	1.1	rWT	FH8	3C protease	23.41	0.22	9.34	0
CUSF-AsPgb1.1-rWT	AsPgb	1.1	rWT	CUSF	3C protease	23.41	0.22	9.25	0
FH8-AsPgb1.1-rWT	AsPgb	1.1	rWT	FH8	DnaK Mix	33.73	0.31	9.17	0
_-AsPgb1.1-rWT	AsPgb	1.1	rWT	_	GSSG	25.05	0.23	9.07	0
_-AsPgb1.1-rWT	AsPgb	1.1	rWT	_	Zn2+	25.05	0.22	8.87	0
CUSF-AsPgb1.1-rWT	AsPgb	1.1	rWT	CUSF	DnaK Mix	36.12	0.32	8.84	0
_-AsPgb1.1-rWT	AsPgb	1.1	rWT	_	Cofactor Mix	25.05	0.22	8.83	0
_-AsPgb1.1-rWT	AsPgb	1.1	rWT	_	TRXB1	25.05	0.21	8.42	0
_-AsPgb1.1-rWT	AsPgb	1.1	rWT	_	Buffer	25.05	0.21	8.23	0
P17-AsPgb1.5-rWT	AsPgb	1.5	rWT	P17	PDI/GSSG	31	0.44	14.12	5.93
_-AsPgb1.5-rWT	AsPgb	1.5	rWT	_	PDI/GSSG	26.04	0.41	15.82	5.92
_-AsPgb1.5-rWT	AsPgb	1.5	rWT	_	3C protease	24.4	0.37	15.17	5.79
P17-AsPgb1.5-rWT	AsPgb	1.5	rWT	P17	GSSG	31	0.43	13.96	4.79
_-AsPgb1.5-rWT	AsPgb	1.5	rWT	_	GSSG	26.04	0.37	14.17	4.66
_-AsPgb1.5-rWT	AsPgb	1.5	rWT	_	DnaK Mix	26.04	0.36	13.77	0

P17-AsPgb1.5-rWT	AsPgb	1.5	rWT	P17	Cofactor Mix	31	0.42	13.7	0
_-AsPgb1.5-rWT	AsPgb	1.5	rWT	_	Cofactor Mix	26.04	0.35	13.53	0
FH8-AsPgb1.5-rWT	AsPgb	1.5	rWT	FH8	PDI/GSSG	34.72	0.47	13.51	0
P17-AsPgb1.5-rWT	AsPgb	1.5	rWT	P17	TRXB1	31	0.42	13.48	0
_-AsPgb1.5-rWT	AsPgb	1.5	rWT	_	Zn2+	26.04	0.35	13.34	0
P17-AsPgb1.5-rWT	AsPgb	1.5	rWT	P17	Zn2+	31	0.41	13.23	0
FH8-AsPgb1.5-rWT	AsPgb	1.5	rWT	FH8	GSSG	34.72	0.46	13.12	0
_-AsPgb1.5-rWT	AsPgb	1.5	rWT	_	TRXB1	26.04	0.34	13.1	0
P17-AsPgb1.5-rWT	AsPgb	1.5	rWT	P17	Buffer	31	0.41	13.09	0
P17-AsPgb1.5-rWT	AsPgb	1.5	rWT	P17	3C protease	24.4	0.31	12.91	0
CUSF-AsPgb1.5-rWT	AsPgb	1.5	rWT	CUSF	PDI/GSSG	37.11	0.47	12.78	0
_-AsPgb1.5-rWT	AsPgb	1.5	rWT	_	Buffer	26.04	0.33	12.69	0
CUSF-AsPgb1.5-rWT	AsPgb	1.5	rWT	CUSF	GSSG	37.11	0.47	12.69	0
FH8-AsPgb1.5-rWT	AsPgb	1.5	rWT	FH8	TRXB1	34.72	0.43	12.4	0
P17-AsPgb1.5-rWT	AsPgb	1.5	rWT	P17	DnaK Mix	31	0.38	12.36	0
FH8-AsPgb1.5-rWT	AsPgb	1.5	rWT	FH8	3C protease	24.4	0.3	12.35	0
FH8-AsPgb1.5-rWT	AsPgb	1.5	rWT	FH8	Buffer	34.72	0.42	12.13	0
CUSF-AsPgb1.5-rWT	AsPgb	1.5	rWT	CUSF	Cofactor Mix	37.11	0.45	12.09	0
CUSF-AsPgb1.5-rWT	AsPgb	1.5	rWT	CUSF	3C protease	24.4	0.29	12.06	0
CUSF-AsPgb1.5-rWT	AsPgb	1.5	rWT	CUSF	TRXB1	37.11	0.44	11.98	0
CUSF-AsPgb1.5-rWT	AsPgb	1.5	rWT	CUSF	Zn2+	37.11	0.44	11.8	0
FH8-AsPgb1.5-rWT	AsPgb	1.5	rWT	FH8	DnaK Mix	34.72	0.41	11.68	0
FH8-AsPgb1.5-rWT	AsPgb	1.5	rWT	FH8	Cofactor Mix	34.72	0.4	11.52	0
CUSF-AsPgb1.5-rWT	AsPgb	1.5	rWT	CUSF	DnaK Mix	37.11	0.42	11.24	0
CUSF-AsPgb1.5-rWT	AsPgb	1.5	rWT	CUSF	Buffer	37.11	0.42	11.18	0
FH8-AsPgb1.5-rWT	AsPgb	1.5	rWT	FH8	Zn2+	34.72	0.28	8.17	0
P17-AsPgb3.1-rWT	AsPgb	3.1	rWT	P17	GSSG	32.23	0.3	9.46	5.79
P17-AsPgb3.1-rWT	AsPgb	3.1	rWT	P17	PDI/GSSG	32.23	0.31	9.67	5.72
P17-AsPgb3.1-rWT	AsPgb	3.1	rWT	P17	Zn2+	32.23	0.31	9.65	5.52
_-AsPgb3.1-rWT	AsPgb	3.1	rWT	_	3C protease	25.63	0.25	9.68	5.21
P17-AsPgb3.1-rWT	AsPgb	3.1	rWT	P17	Cofactor Mix	32.23	0.33	10.23	5.2
P17-AsPgb3.1-rWT	AsPgb	3.1	rWT	P17	TRXB1	32.23	0.3	9.33	0
FH8-AsPgb3.1-rWT	AsPgb	3.1	rWT	FH8	3C protease	25.63	0.23	9.06	0
P17-AsPgb3.1-rWT	AsPgb	3.1	rWT	P17	DnaK Mix	32.23	0.28	8.66	0
P17-AsPgb3.1-rWT	AsPgb	3.1	rWT	P17	Buffer	32.23	0.28	8.59	0
CUSF-AsPgb3.1-rWT	AsPgb	3.1	rWT	CUSF	3C protease	25.63	0.22	8.51	0
FH8-AsPgb3.1-rWT	AsPgb	3.1	rWT	FH8	Cofactor Mix	35.95	0.3	8.44	0
FH8-AsPgb3.1-rWT	AsPgb	3.1	rWT	FH8	Zn2+	35.95	0.3	8.21	0
FH8-AsPgb3.1-rWT	AsPgb	3.1	rWT	FH8	PDI/GSSG	35.95	0.28	7.93	0
_-AsPgb3.1-rWT	AsPgb	3.1	rWT	_	PDI/GSSG	27.27	0.22	7.92	0
FH8-AsPgb3.1-rWT	AsPgb	3.1	rWT	FH8	GSSG	35.95	0.28	7.87	0
FH8-AsPgb3.1-rWT	AsPgb	3.1	rWT	FH8	TRXB1	35.95	0.28	7.85	0

_-AsPgb3.1-rWT	AsPgb	3.1	rWT	_	Cofactor Mix	27.27	0.21	7.81	0
FH8-AsPgb3.1-rWT	AsPgb	3.1	rWT	FH8	Buffer	35.95	0.28	7.8	0
_-AsPgb3.1-rWT	AsPgb	3.1	rWT	_	GSSG	27.27	0.21	7.7	0
_-AsPgb3.1-rWT	AsPgb	3.1	rWT	_	Zn2+	27.27	0.21	7.62	0
FH8-AsPgb3.1-rWT	AsPgb	3.1	rWT	FH8	DnaK Mix	35.95	0.27	7.53	0
CUSF-AsPgb3.1-rWT	AsPgb	3.1	rWT	CUSF	Zn2+	38.34	0.28	7.38	0
_-AsPgb3.1-rWT	AsPgb	3.1	rWT	_	DnaK Mix	27.27	0.2	7.36	0
CUSF-AsPgb3.1-rWT	AsPgb	3.1	rWT	CUSF	PDI/GSSG	38.34	0.28	7.26	0
CUSF-AsPgb3.1-rWT	AsPgb	3.1	rWT	CUSF	Cofactor Mix	38.34	0.28	7.24	0
_-AsPgb3.1-rWT	AsPgb	3.1	rWT	_	TRXB1	27.27	0.2	7.2	0
_-AsPgb3.1-rWT	AsPgb	3.1	rWT	_	Buffer	27.27	0.19	7.06	0
CUSF-AsPgb3.1-rWT	AsPgb	3.1	rWT	CUSF	GSSG	38.34	0.27	7.03	0
CUSF-AsPgb3.1-rWT	AsPgb	3.1	rWT	CUSF	TRXB1	38.34	0.27	6.99	0
CUSF-AsPgb3.1-rWT	AsPgb	3.1	rWT	CUSF	DnaK Mix	38.34	0.26	6.7	0
CUSF-AsPgb3.1-rWT	AsPgb	3.1	rWT	CUSF	Buffer	38.34	0.25	6.58	0
P17-AsPgb3.1-rWT	AsPgb	3.1	rWT	P17	3C protease	25.63	0.11	4.4	0
CUSF-AsPgb1.5-C84A	AsPgb	1.5	C84A	CUSF	PDI/GSSG	37.08	0.5	13.46	6.16
CUSF-AsPgb1.5-C84A	AsPgb	1.5	C84A	CUSF	Cofactor Mix	37.08	0.5	13.53	5.99
CUSF-AsPgb1.5-C84A	AsPgb	1.5	C84A	CUSF	GSSG	37.08	0.5	13.49	5.96
P17-AsPgb1.5-C84A	AsPgb	1.5	C84A	P17	PDI/GSSG	30.97	0.43	13.91	3.93
P17-AsPgb1.5-C84A	AsPgb	1.5	C84A	P17	Cofactor Mix	30.97	0.42	13.41	3.45
P17-AsPgb1.5-C84A	AsPgb	1.5	C84A	P17	GSSG	30.97	0.41	13.09	0
P17-AsPgb1.5-C84A	AsPgb	1.5	C84A	P17	TRXB1	30.97	0.4	13.05	0
CUSF-AsPgb1.5-C84A	AsPgb	1.5	C84A	CUSF	TRXB1	37.08	0.48	12.98	0
CUSF-AsPgb1.5-C84A	AsPgb	1.5	C84A	CUSF	Zn2+	37.08	0.48	12.95	0
P17-AsPgb1.5-C84A	AsPgb	1.5	C84A	P17	Buffer	30.97	0.4	12.89	0
P17-AsPgb1.5-C84A	AsPgb	1.5	C84A	P17	Zn2+	30.97	0.4	12.88	0
CUSF-AsPgb1.5-C84A	AsPgb	1.5	C84A	CUSF	Buffer	37.08	0.47	12.75	0
FH8-AsPgb1.5-C84A	AsPgb	1.5	C84A	FH8	Cofactor Mix	34.69	0.42	12.02	0
CUSF-AsPgb1.5-C84A	AsPgb	1.5	C84A	CUSF	DnaK Mix	37.08	0.44	11.94	0
P17-AsPgb1.5-C84A	AsPgb	1.5	C84A	P17	3C protease	24.36	0.29	11.88	0
FH8-AsPgb1.5-C84A	AsPgb	1.5	C84A	FH8	PDI/GSSG	34.69	0.41	11.86	0
CUSF-AsPgb1.5-C84A	AsPgb	1.5	C84A	CUSF	3C protease	24.36	0.29	11.85	0
P17-AsPgb1.5-C84A	AsPgb	1.5	C84A	P17	DnaK Mix	30.97	0.37	11.83	0
FH8-AsPgb1.5-C84A	AsPgb	1.5	C84A	FH8	TRXB1	34.69	0.4	11.56	0
FH8-AsPgb1.5-C84A	AsPgb	1.5	C84A	FH8	Zn2+	34.69	0.4	11.55	0
FH8-AsPgb1.5-C84A	AsPgb	1.5	C84A	FH8	GSSG	34.69	0.4	11.47	0
FH8-AsPgb1.5-C84A	AsPgb	1.5	C84A	FH8	Buffer	34.69	0.4	11.46	0
FH8-AsPgb1.5-C84A	AsPgb	1.5	C84A	FH8	DnaK Mix	34.69	0.37	10.77	0
FH8-AsPgb1.5-C84A	AsPgb	1.5	C84A	FH8	3C protease	24.36	0.26	10.66	0
_-AsPgb1.5-C84A	AsPgb	1.5	C84A	_	DnaK Mix	26.01	0.26	9.89	0
_-AsPgb1.5-C84A	AsPgb	1.5	C84A	_	Cofactor Mix	26.01	0.24	9.39	0

_-AsPgb1.5-C84A	AsPgb	1.5	C84A	_	3C protease	24.36	0.23	9.39	0
_-AsPgb1.5-C84A	AsPgb	1.5	C84A	_	Buffer	26.01	0.23	8.93	0
_-AsPgb1.5-C84A	AsPgb	1.5	C84A	_	PDI/GSSG	26.01	0.23	8.75	0
_-AsPgb1.5-C84A	AsPgb	1.5	C84A	_	TRXB1	26.01	0.22	8.63	0
_-AsPgb1.5-C84A	AsPgb	1.5	C84A	_	Zn2+	26.01	0.22	8.48	0
_-AsPgb1.5-C84A	AsPgb	1.5	C84A	_	GSSG	26.01	0.21	8.11	0
CUSF-AsPgb1.1-C70A	AsPgb	1.1	C70A	CUSF	PDI/GSSG	36.09	0.46	12.77	4.19
CUSF-AsPgb1.1-C70A	AsPgb	1.1	C70A	CUSF	GSSG	36.09	0.47	12.91	3.69
P17-AsPgb1.1-C70A	AsPgb	1.1	C70A	P17	PDI/GSSG	29.98	0.41	13.55	1.52
P17-AsPgb1.1-C70A	AsPgb	1.1	C70A	P17	Cofactor Mix	29.98	0.38	12.83	1.42
P17-AsPgb1.1-C70A	AsPgb	1.1	C70A	P17	GSSG	29.98	0.4	13.32	1.35
_-AsPgb1.1-C70A	AsPgb	1.1	C70A	_	DnaK Mix	25.02	0.32	12.61	0
CUSF-AsPgb1.1-C70A	AsPgb	1.1	C70A	CUSF	Cofactor Mix	36.09	0.45	12.55	0
P17-AsPgb1.1-C70A	AsPgb	1.1	C70A	P17	Zn2+	29.98	0.38	12.52	0
_-AsPgb1.1-C70A	AsPgb	1.1	C70A	_	3C protease	23.38	0.29	12.52	0
P17-AsPgb1.1-C70A	AsPgb	1.1	C70A	P17	Buffer	29.98	0.37	12.4	0
P17-AsPgb1.1-C70A	AsPgb	1.1	C70A	P17	TRXB1	29.98	0.36	12.1	0
CUSF-AsPgb1.1-C70A	AsPgb	1.1	C70A	CUSF	Zn2+	36.09	0.43	12.04	0
_-AsPgb1.1-C70A	AsPgb	1.1	C70A	_	PDI/GSSG	25.02	0.3	11.95	0
CUSF-AsPgb1.1-C70A	AsPgb	1.1	C70A	CUSF	Buffer	36.09	0.42	11.71	0
CUSF-AsPgb1.1-C70A	AsPgb	1.1	C70A	CUSF	TRXB1	36.09	0.42	11.52	0
CUSF-AsPgb1.1-C70A	AsPgb	1.1	C70A	CUSF	DnaK Mix	36.09	0.4	11.13	0
P17-AsPgb1.1-C70A	AsPgb	1.1	C70A	P17	DnaK Mix	29.98	0.33	10.86	0
_-AsPgb1.1-C70A	AsPgb	1.1	C70A	_	GSSG	25.02	0.26	10.38	0
CUSF-AsPgb1.1-C70A	AsPgb	1.1	C70A	CUSF	3C protease	23.38	0.24	10.3	0
P17-AsPgb1.1-C70A	AsPgb	1.1	C70A	P17	3C protease	23.38	0.24	10.07	0
FH8-AsPgb1.1-C70A	AsPgb	1.1	C70A	FH8	TRXB1	33.7	0.33	9.88	0
FH8-AsPgb1.1-C70A	AsPgb	1.1	C70A	FH8	Cofactor Mix	33.7	0.32	9.54	0
FH8-AsPgb1.1-C70A	AsPgb	1.1	C70A	FH8	Zn2+	33.7	0.32	9.35	0
FH8-AsPgb1.1-C70A	AsPgb	1.1	C70A	FH8	Buffer	33.7	0.31	9.31	0
FH8-AsPgb1.1-C70A	AsPgb	1.1	C70A	FH8	PDI/GSSG	33.7	0.31	9.16	0
FH8-AsPgb1.1-C70A	AsPgb	1.1	C70A	FH8	3C protease	23.38	0.21	9.04	0
FH8-AsPgb1.1-C70A	AsPgb	1.1	C70A	FH8	DnaK Mix	33.7	0.3	8.86	0
_-AsPgb1.1-C70A	AsPgb	1.1	C70A	_	Cofactor Mix	25.02	0.21	8.53	0
_-AsPgb1.1-C70A	AsPgb	1.1	C70A	_	Zn2+	25.02	0.21	8.25	0
_-AsPgb1.1-C70A	AsPgb	1.1	C70A	_	Buffer	25.02	0.2	8.12	0
_-AsPgb1.1-C70A	AsPgb	1.1	C70A	_	TRXB1	25.02	0.19	7.53	0
FH8-AsPgb1.1-C70A	AsPgb	1.1	C70A	FH8	GSSG	33.7	0.25	7.34	0
_-AsPgb3.1-C161A	AsPgb	3.1	C161A	_	3C protease	25.6	0.32	12.57	6.62
_-AsPgb3.1-C161A	AsPgb	3.1	C161A	_	TRXB1	27.24	0.28	10.42	6.13
_-AsPgb3.1-C161A	AsPgb	3.1	C161A	_	Cofactor Mix	27.24	0.31	11.29	5.79
P17-AsPgb3.1-C161A	AsPgb	3.1	C161A	P17	3C protease	25.6	0.27	10.54	5.47

_-AsPgb3.1-C161A	AsPgb	3.1	C161A	_	Zn2+	27.24	0.31	11.23	5.37
_-AsPgb3.1-C161A	AsPgb	3.1	C161A	_	Buffer	27.24	0.28	10.37	0
_-AsPgb3.1-C161A	AsPgb	3.1	C161A	_	PDI/GSSG	27.24	0.28	10.13	0
_-AsPgb3.1-C161A	AsPgb	3.1	C161A	_	DnaK Mix	27.24	0.28	10.12	0
P17-AsPgb3.1-C161A	AsPgb	3.1	C161A	P17	Cofactor Mix	32.2	0.33	10.1	0
CUSF-AsPgb3.1-C161A	AsPgb	3.1	C161A	CUSF	3C protease	25.6	0.25	9.9	0
_-AsPgb3.1-C161A	AsPgb	3.1	C161A	_	GSSG	27.24	0.27	9.77	0
P17-AsPgb3.1-C161A	AsPgb	3.1	C161A	P17	Zn2+	32.2	0.31	9.68	0
CUSF-AsPgb3.1-C161A	AsPgb	3.1	C161A	CUSF	Cofactor Mix	38.31	0.37	9.62	0
P17-AsPgb3.1-C161A	AsPgb	3.1	C161A	P17	TRXB1	32.2	0.29	9.13	0
CUSF-AsPgb3.1-C161A	AsPgb	3.1	C161A	CUSF	Zn2+	38.31	0.35	9.12	0
P17-AsPgb3.1-C161A	AsPgb	3.1	C161A	P17	PDI/GSSG	32.2	0.29	9.07	0
P17-AsPgb3.1-C161A	AsPgb	3.1	C161A	P17	GSSG	32.2	0.29	9.06	0
P17-AsPgb3.1-C161A	AsPgb	3.1	C161A	P17	Buffer	32.2	0.29	9	0
CUSF-AsPgb3.1-C161A	AsPgb	3.1	C161A	CUSF	TRXB1	38.31	0.34	8.85	0
CUSF-AsPgb3.1-C161A	AsPgb	3.1	C161A	CUSF	Buffer	38.31	0.33	8.66	0
P17-AsPgb3.1-C161A	AsPgb	3.1	C161A	P17	DnaK Mix	32.2	0.28	8.61	0
FH8-AsPgb3.1-C161A	AsPgb	3.1	C161A	FH8	3C protease	25.6	0.22	8.6	0
CUSF-AsPgb3.1-C161A	AsPgb	3.1	C161A	CUSF	DnaK Mix	38.31	0.33	8.52	0
CUSF-AsPgb3.1-C161A	AsPgb	3.1	C161A	CUSF	GSSG	38.31	0.32	8.26	0
CUSF-AsPgb3.1-C161A	AsPgb	3.1	C161A	CUSF	PDI/GSSG	38.31	0.31	8.18	0
FH8-AsPgb3.1-C161A	AsPgb	3.1	C161A	FH8	Cofactor Mix	35.92	0.29	8.11	0
FH8-AsPgb3.1-C161A	AsPgb	3.1	C161A	FH8	Zn2+	35.92	0.27	7.53	0
FH8-AsPgb3.1-C161A	AsPgb	3.1	C161A	FH8	TRXB1	35.92	0.27	7.47	0
FH8-AsPgb3.1-C161A	AsPgb	3.1	C161A	FH8	Buffer	35.92	0.26	7.22	0
FH8-AsPgb3.1-C161A	AsPgb	3.1	C161A	FH8	DnaK Mix	35.92	0.26	7.21	0
FH8-AsPgb3.1-C161A	AsPgb	3.1	C161A	FH8	PDI/GSSG	35.92	0.26	7.15	0
FH8-AsPgb3.1-C161A	AsPgb	3.1	C161A	FH8	GSSG	35.92	0.24	6.81	0
CUSF-BvPgb1.2-rWT	BvPgb	1.2	rWT	CUSF	PDI/GSSG	38.03	0.4	10.59	6.69
FH8-BvPgb1.2-rWT	BvPgb	1.2	rWT	FH8	Zn2+	35.64	0.38	10.55	6.52
CUSF-BvPgb1.2-rWT	BvPgb	1.2	rWT	CUSF	Zn2+	38.03	0.4	10.59	6.49
_-BvPgb1.2-rWT	BvPgb	1.2	rWT	_	PDI/GSSG	26.96	0.31	11.47	6.4
FH8-BvPgb1.2-rWT	BvPgb	1.2	rWT	FH8	PDI/GSSG	35.64	0.38	10.77	6.35
P17-BvPgb1.2-rWT	BvPgb	1.2	rWT	P17	PDI/GSSG	31.92	0.35	11.12	6.32
P17-BvPgb1.2-rWT	BvPgb	1.2	rWT	P17	GSSG	31.92	0.34	10.76	6.12
_-BvPgb1.2-rWT	BvPgb	1.2	rWT	_	3C protease	25.32	0.27	10.81	6.01
CUSF-BvPgb1.2-rWT	BvPgb	1.2	rWT	CUSF	3C protease	25.32	0.28	10.96	5.92
_-BvPgb1.2-rWT	BvPgb	1.2	rWT	_	GSSG	26.96	0.29	10.8	5.61
P17-BvPgb1.2-rWT	BvPgb	1.2	rWT	P17	Cofactor Mix	31.92	0.33	10.47	0
CUSF-BvPgb1.2-rWT	BvPgb	1.2	rWT	CUSF	Cofactor Mix	38.03	0.4	10.44	0
FH8-BvPgb1.2-rWT	BvPgb	1.2	rWT	FH8	GSSG	35.64	0.37	10.3	0
P17-BvPgb1.2-rWT	BvPgb	1.2	rWT	P17	3C protease	25.32	0.26	10.21	0

FH8-BvPgb1.2-rWT	BvPgb	1.2	rWT	FH8	Cofactor Mix	35.64	0.36	10.21	0
_-BvPgb1.2-rWT	BvPgb	1.2	rWT	_	Cofactor Mix	26.96	0.27	10.16	0
CUSF-BvPgb1.2-rWT	BvPgb	1.2	rWT	CUSF	GSSG	38.03	0.38	9.89	0
P17-BvPgb1.2-rWT	BvPgb	1.2	rWT	P17	TRXB1	31.92	0.31	9.81	0
CUSF-BvPgb1.2-rWT	BvPgb	1.2	rWT	CUSF	TRXB1	38.03	0.37	9.63	0
_-BvPgb1.2-rWT	BvPgb	1.2	rWT	_	Zn2+	26.96	0.26	9.57	0
ZZ-BvPgb1.2-rWT	BvPgb	1.2	rWT	ZZ	GSSG	41.31	0.4	9.56	0
FH8-BvPgb1.2-rWT	BvPgb	1.2	rWT	FH8	TRXB1	35.64	0.34	9.55	0
TRX-BvPgb1.2-rWT	BvPgb	1.2	rWT	TRX	PDI/GSSG	39.78	0.38	9.43	0
ZZ-BvPgb1.2-rWT	BvPgb	1.2	rWT	ZZ	PDI/GSSG	41.31	0.39	9.43	0
_-BvPgb1.2-rWT	BvPgb	1.2	rWT	_	TRXB1	26.96	0.25	9.41	0
ZZ-BvPgb1.2-rWT	BvPgb	1.2	rWT	ZZ	Zn2+	41.31	0.39	9.4	0
ZZ-BvPgb1.2-rWT	BvPgb	1.2	rWT	ZZ	Cofactor Mix	41.31	0.39	9.35	0
TRX-BvPgb1.2-rWT	BvPgb	1.2	rWT	TRX	GSSG	39.78	0.36	9.14	0
_-BvPgb1.2-rWT	BvPgb	1.2	rWT	_	Buffer	26.96	0.25	9.11	0
TRX-BvPgb1.2-rWT	BvPgb	1.2	rWT	TRX	3C protease	25.32	0.23	9.11	0
_-BvPgb1.2-rWT	BvPgb	1.2	rWT	_	DnaK Mix	26.96	0.24	8.88	0
TRX-BvPgb1.2-rWT	BvPgb	1.2	rWT	TRX	Cofactor Mix	39.78	0.35	8.87	0
FH8-BvPgb1.2-rWT	BvPgb	1.2	rWT	FH8	Buffer	35.64	0.31	8.8	0
TRX-BvPgb1.2-rWT	BvPgb	1.2	rWT	TRX	Zn2+	39.78	0.35	8.8	0
ZZ-BvPgb1.2-rWT	BvPgb	1.2	rWT	ZZ	3C protease	25.32	0.22	8.65	0
ZZ-BvPgb1.2-rWT	BvPgb	1.2	rWT	ZZ	TRXB1	41.31	0.36	8.62	0
P17-BvPgb1.2-rWT	BvPgb	1.2	rWT	P17	Buffer	31.92	0.27	8.43	0
TRX-BvPgb1.2-rWT	BvPgb	1.2	rWT	TRX	TRXB1	39.78	0.33	8.38	0
CUSF-BvPgb1.2-rWT	BvPgb	1.2	rWT	CUSF	Buffer	38.03	0.31	8.25	0
CUSF-BvPgb1.2-rWT	BvPgb	1.2	rWT	CUSF	DnaK Mix	38.03	0.31	8.22	0
ZZ-BvPgb1.2-rWT	BvPgb	1.2	rWT	ZZ	Buffer	41.31	0.34	8.16	0
SNUT-BvPgb1.2-rWT	BvPgb	1.2	rWT	SNUT	3C protease	25.32	0.21	8.12	0
FH8-BvPgb1.2-rWT	BvPgb	1.2	rWT	FH8	DnaK Mix	35.64	0.29	8.08	0
P17-BvPgb1.2-rWT	BvPgb	1.2	rWT	P17	DnaK Mix	31.92	0.26	8.04	0
SNUT-BvPgb1.2-rWT	BvPgb	1.2	rWT	SNUT	GSSG	44.86	0.35	7.74	0
SNUT-BvPgb1.2-rWT	BvPgb	1.2	rWT	SNUT	Cofactor Mix	44.86	0.35	7.72	0
TRX-BvPgb1.2-rWT	BvPgb	1.2	rWT	TRX	Buffer	39.78	0.31	7.69	0
SNUT-BvPgb1.2-rWT	BvPgb	1.2	rWT	SNUT	PDI/GSSG	44.86	0.34	7.66	0
ZZ-BvPgb1.2-rWT	BvPgb	1.2	rWT	ZZ	DnaK Mix	41.31	0.31	7.55	0
SNUT-BvPgb1.2-rWT	BvPgb	1.2	rWT	SNUT	Zn2+	44.86	0.34	7.47	0
TRX-BvPgb1.2-rWT	BvPgb	1.2	rWT	TRX	DnaK Mix	39.78	0.29	7.33	0
P17-BvPgb1.2-rWT	BvPgb	1.2	rWT	P17	Zn2+	31.92	0.23	7.31	0
SNUT-BvPgb1.2-rWT	BvPgb	1.2	rWT	SNUT	TRXB1	44.86	0.32	7.07	0
SNUT-BvPgb1.2-rWT	BvPgb	1.2	rWT	SNUT	Buffer	44.86	0.3	6.78	0
SNUT-BvPgb1.2-rWT	BvPgb	1.2	rWT	SNUT	DnaK Mix	44.86	0.28	6.18	0
SUMO-BvPgb1.2-rWT	BvPgb	1.2	rWT	SUMO	3C protease	25.32	0.13	5.32	0

SUMO-BvPgb1.2-rWT	BvPgb	1.2	rWT	SUMO	PDI/GSSG	39.61	0.2	5.11	0
SUMO-BvPgb1.2-rWT	BvPgb	1.2	rWT	SUMO	Zn2+	39.61	0.2	5.05	0
SUMO-BvPgb1.2-rWT	BvPgb	1.2	rWT	SUMO	Cofactor Mix	39.61	0.2	4.97	0
SUMO-BvPgb1.2-rWT	BvPgb	1.2	rWT	SUMO	GSSG	39.61	0.19	4.82	0
SUMO-BvPgb1.2-rWT	BvPgb	1.2	rWT	SUMO	Buffer	39.61	0.16	4.01	0
SUMO-BvPgb1.2-rWT	BvPgb	1.2	rWT	SUMO	DnaK Mix	39.61	0.16	4	0
FH8-BvPgb1.2-rWT	BvPgb	1.2	rWT	FH8	3C protease	25.32	0.08	3.08	0
SUMO-BvPgb1.2-rWT	BvPgb	1.2	rWT	SUMO	TRXB1	39.61	0.09	2.23	0
_-BvPgb1.2-C86A	BvPgb	1.2	C86A	_	3C protease	25.29	0.39	15.43	7.14
CUSF-BvPgb1.2-C86A	BvPgb	1.2	C86A	CUSF	3C protease	25.29	0.3	11.89	7.07
CUSF-BvPgb1.2-C86A	BvPgb	1.2	C86A	CUSF	GSSG	38	0.45	11.73	6.85
_-BvPgb1.2-C86A	BvPgb	1.2	C86A	_	DnaK Mix	26.93	0.38	13.94	6.79
_-BvPgb1.2-C86A	BvPgb	1.2	C86A	_	Buffer	26.93	0.39	14.31	6.3
_-BvPgb1.2-C86A	BvPgb	1.2	C86A	_	PDI/GSSG	26.93	0.38	14.21	6.26
_-BvPgb1.2-C86A	BvPgb	1.2	C86A	_	TRXB1	26.93	0.39	14.46	6.15
_-BvPgb1.2-C86A	BvPgb	1.2	C86A	_	GSSG	26.93	0.38	14.18	5.57
_-BvPgb1.2-C86A	BvPgb	1.2	C86A	_	Zn2+	26.93	0.39	14.49	5.43
_-BvPgb1.2-C86A	BvPgb	1.2	C86A	_	Cofactor Mix	26.93	0.36	13.32	5.06
CUSF-BvPgb1.2-C86A	BvPgb	1.2	C86A	CUSF	PDI/GSSG	38	0.44	11.67	0
TRX-BvPgb1.2-C86A	BvPgb	1.2	C86A	TRX	Zn2+	39.75	0.44	10.98	0
CUSF-BvPgb1.2-C86A	BvPgb	1.2	C86A	CUSF	Cofactor Mix	38	0.42	10.97	0
FH8-BvPgb1.2-C86A	BvPgb	1.2	C86A	FH8	PDI/GSSG	35.61	0.39	10.95	0
SNUT-BvPgb1.2-C86A	BvPgb	1.2	C86A	SNUT	3C protease	25.29	0.28	10.92	0
TRX-BvPgb1.2-C86A	BvPgb	1.2	C86A	TRX	PDI/GSSG	39.75	0.43	10.9	0
FH8-BvPgb1.2-C86A	BvPgb	1.2	C86A	FH8	GSSG	35.61	0.39	10.89	0
TRX-BvPgb1.2-C86A	BvPgb	1.2	C86A	TRX	3C protease	25.29	0.27	10.8	0
SNUT-BvPgb1.2-C86A	BvPgb	1.2	C86A	SNUT	Cofactor Mix	44.83	0.48	10.64	0
TRX-BvPgb1.2-C86A	BvPgb	1.2	C86A	TRX	GSSG	39.75	0.42	10.63	0
SNUT-BvPgb1.2-C86A	BvPgb	1.2	C86A	SNUT	Zn2+	44.83	0.48	10.6	0
FH8-BvPgb1.2-C86A	BvPgb	1.2	C86A	FH8	Zn2+	35.61	0.38	10.56	0
TRX-BvPgb1.2-C86A	BvPgb	1.2	C86A	TRX	Cofactor Mix	39.75	0.42	10.54	0
CUSF-BvPgb1.2-C86A	BvPgb	1.2	C86A	CUSF	TRXB1	38	0.39	10.36	0
SNUT-BvPgb1.2-C86A	BvPgb	1.2	C86A	SNUT	TRXB1	44.83	0.46	10.31	0
SNUT-BvPgb1.2-C86A	BvPgb	1.2	C86A	SNUT	GSSG	44.83	0.46	10.31	0
SUMO-BvPgb1.2-C86A	BvPgb	1.2	C86A	SUMO	3C protease	25.29	0.26	10.29	0
TRX-BvPgb1.2-C86A	BvPgb	1.2	C86A	TRX	TRXB1	39.75	0.41	10.24	0
ZZ-BvPgb1.2-C86A	BvPgb	1.2	C86A	ZZ	TRXB1	41.28	0.42	10.14	0
SUMO-BvPgb1.2-C86A	BvPgb	1.2	C86A	SUMO	Zn2+	39.58	0.4	10.1	0
SNUT-BvPgb1.2-C86A	BvPgb	1.2	C86A	SNUT	PDI/GSSG	44.83	0.45	10.09	0
SNUT-BvPgb1.2-C86A	BvPgb	1.2	C86A	SNUT	Buffer	44.83	0.45	10.08	0
P17-BvPgb1.2-C86A	BvPgb	1.2	C86A	P17	3C protease	25.29	0.25	10	0
SUMO-BvPgb1.2-C86A	BvPgb	1.2	C86A	SUMO	PDI/GSSG	39.58	0.39	9.8	0

P17-BvPgb1.2-C86A	BvPgb	1.2	C86A	P17	PDI/GSSG	31.89	0.31	9.69	0
CUSF-BvPgb1.2-C86A	BvPgb	1.2	C86A	CUSF	Buffer	38	0.37	9.66	0
ZZ-BvPgb1.2-C86A	BvPgb	1.2	C86A	ZZ	PDI/GSSG	41.28	0.4	9.66	0
ZZ-BvPgb1.2-C86A	BvPgb	1.2	C86A	ZZ	Buffer	41.28	0.4	9.61	0
P17-BvPgb1.2-C86A	BvPgb	1.2	C86A	P17	Zn2+	31.89	0.31	9.58	0
TRX-BvPgb1.2-C86A	BvPgb	1.2	C86A	TRX	Buffer	39.75	0.38	9.58	0
FH8-BvPgb1.2-C86A	BvPgb	1.2	C86A	FH8	Cofactor Mix	35.61	0.34	9.57	0
FH8-BvPgb1.2-C86A	BvPgb	1.2	C86A	FH8	TRXB1	35.61	0.34	9.56	0
ZZ-BvPgb1.2-C86A	BvPgb	1.2	C86A	ZZ	DnaK Mix	41.28	0.39	9.47	0
SUMO-BvPgb1.2-C86A	BvPgb	1.2	C86A	SUMO	TRXB1	39.58	0.37	9.35	0
P17-BvPgb1.2-C86A	BvPgb	1.2	C86A	P17	Cofactor Mix	31.89	0.3	9.31	0
P17-BvPgb1.2-C86A	BvPgb	1.2	C86A	P17	GSSG	31.89	0.3	9.29	0
SUMO-BvPgb1.2-C86A	BvPgb	1.2	C86A	SUMO	GSSG	39.58	0.37	9.24	0
CUSF-BvPgb1.2-C86A	BvPgb	1.2	C86A	CUSF	DnaK Mix	38	0.35	9.16	0
P17-BvPgb1.2-C86A	BvPgb	1.2	C86A	P17	TRXB1	31.89	0.29	9	0
FH8-BvPgb1.2-C86A	BvPgb	1.2	C86A	FH8	Buffer	35.61	0.32	8.88	0
SNUT-BvPgb1.2-C86A	BvPgb	1.2	C86A	SNUT	DnaK Mix	44.83	0.4	8.87	0
FH8-BvPgb1.2-C86A	BvPgb	1.2	C86A	FH8	DnaK Mix	35.61	0.31	8.74	0
ZZ-BvPgb1.2-C86A	BvPgb	1.2	C86A	ZZ	GSSG	41.28	0.36	8.66	0
TRX-BvPgb1.2-C86A	BvPgb	1.2	C86A	TRX	DnaK Mix	39.75	0.34	8.56	0
SUMO-BvPgb1.2-C86A	BvPgb	1.2	C86A	SUMO	Buffer	39.58	0.33	8.43	0
ZZ-BvPgb1.2-C86A	BvPgb	1.2	C86A	ZZ	Cofactor Mix	41.28	0.35	8.4	0
SUMO-BvPgb1.2-C86A	BvPgb	1.2	C86A	SUMO	DnaK Mix	39.58	0.32	8.17	0
P17-BvPgb1.2-C86A	BvPgb	1.2	C86A	P17	DnaK Mix	31.89	0.24	7.57	0
P17-BvPgb1.2-C86A	BvPgb	1.2	C86A	P17	Buffer	31.89	0.22	6.96	0
SUMO-BvPgb1.2-C86A	BvPgb	1.2	C86A	SUMO	Cofactor Mix	39.58	0.24	5.95	0
CUSF-BvPgb1.2-C86A	BvPgb	1.2	C86A	CUSF	Zn2+	38	0.18	4.65	0
ZZ-BvPgb1.2-C86A	BvPgb	1.2	C86A	ZZ	Zn2+	41.28	0.19	4.5	0
ZZ-BvPgb1.2-C86A	BvPgb	1.2	C86A	ZZ	3C protease	25.29	0.05	1.87	0
FH8-BvPgb1.2-C86A	BvPgb	1.2	C86A	FH8	3C protease	25.29	0.01	0.43	0
