# Supplementary figures and images for: Digital Microfluidics-Driven Cell-Free Protein Synthesis Platform Reveals Expression and Stability Determinants for Phytoglobins and Cysteine-to-Alanine Substituted Variants
Source: Antioxidants (Basel). 2025 Oct 31;14(11):1317. doi: 10.3390/antiox14111317 (PMC12649544; doi:10.3390/antiox14111317)

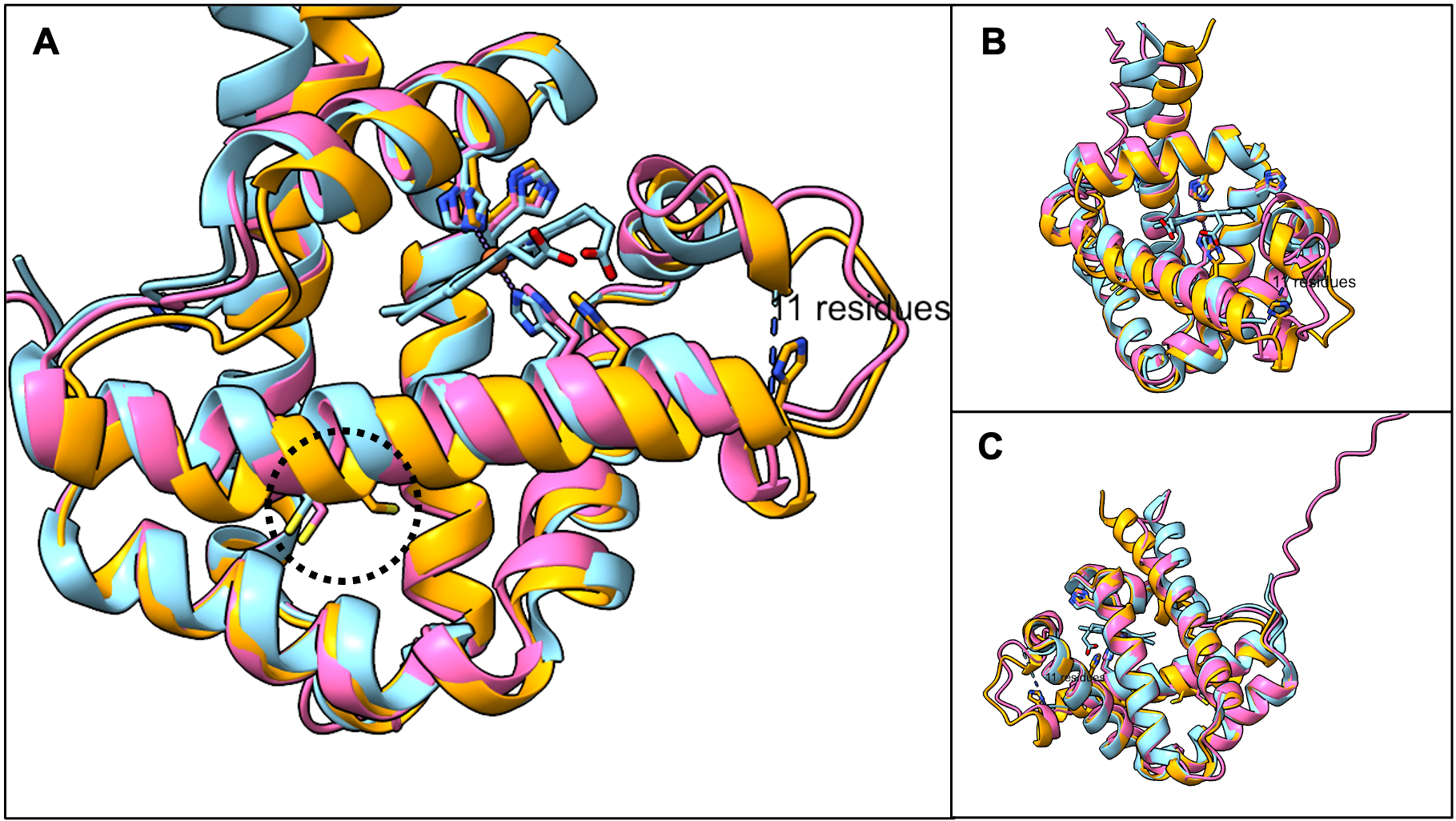

Supplement: Supplementary file 1 [file antioxidants-14-01317-s001.zip › Fig1.png]

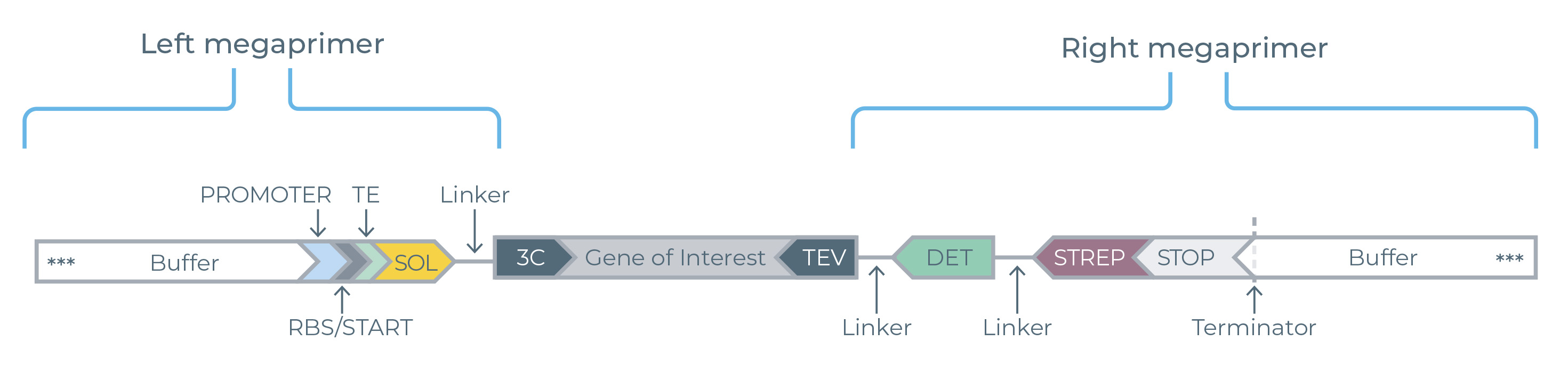

Supplement: Supplementary file 1 [file antioxidants-14-01317-s001.zip › Fig2.jpg]

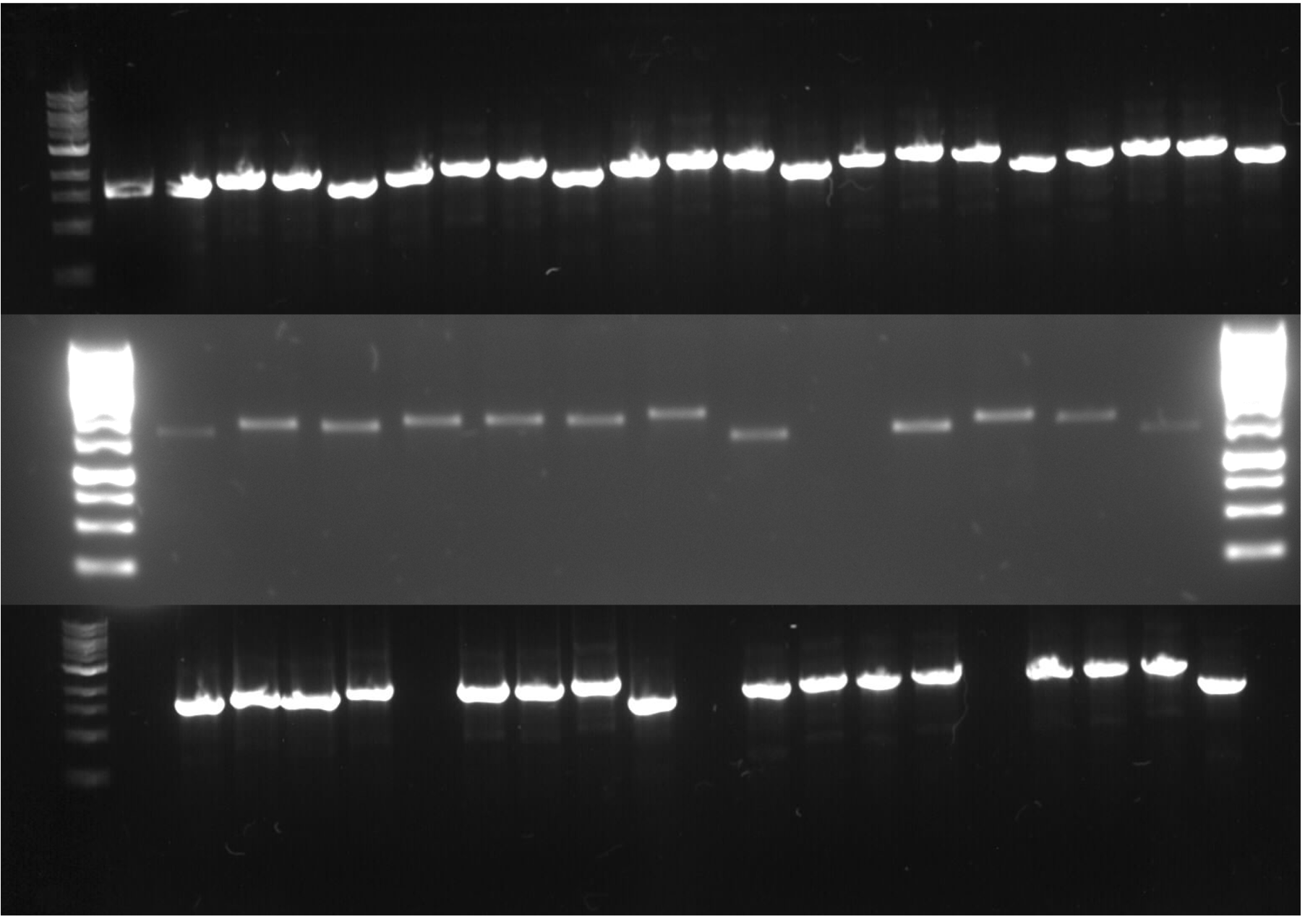

Supplement: Supplementary file 1 [file antioxidants-14-01317-s001.zip › Fig3.png]

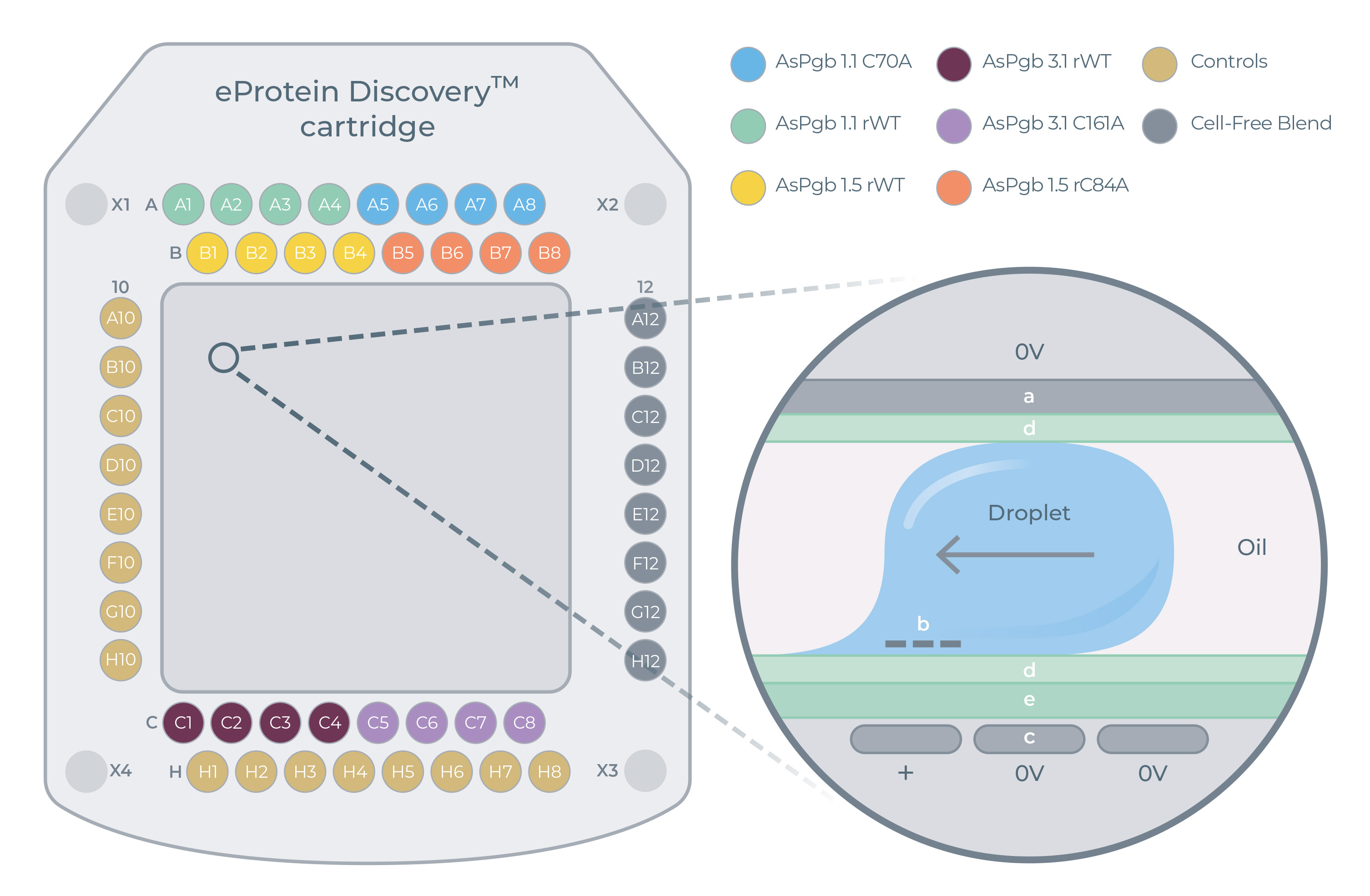

Supplement: Supplementary file 1 [file antioxidants-14-01317-s001.zip › Fig4.jpg]

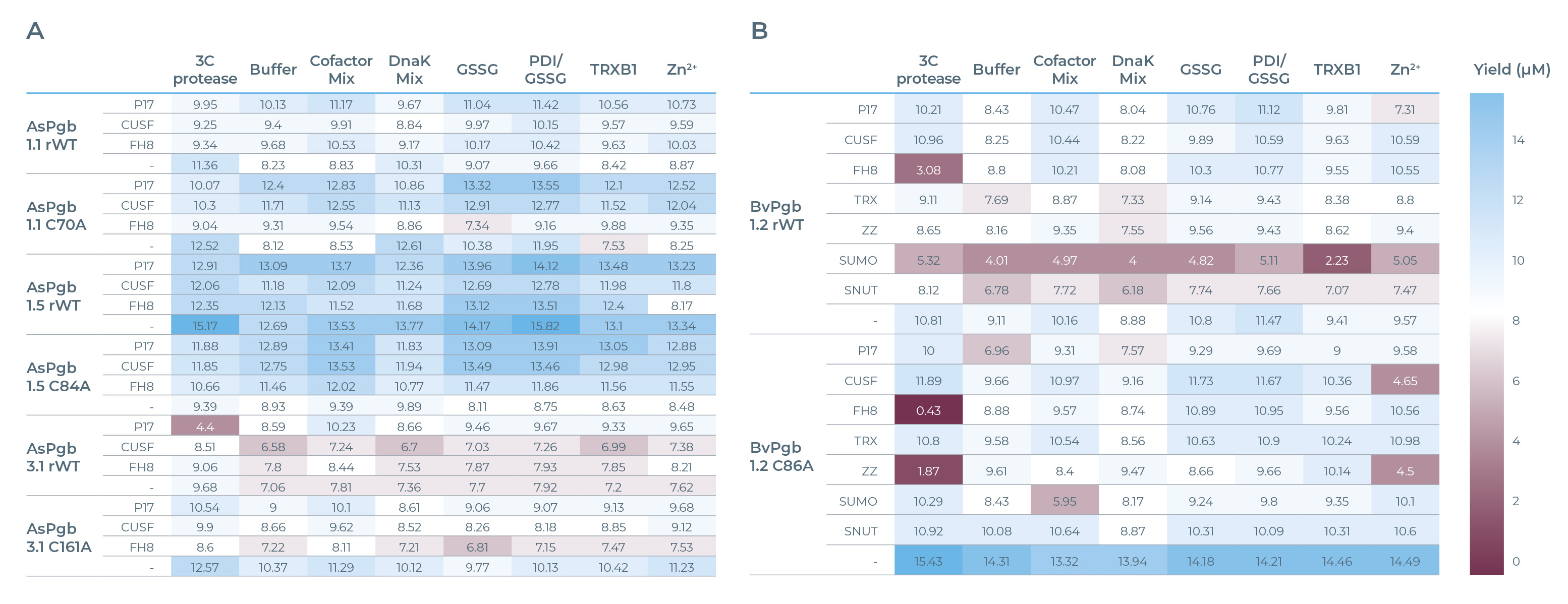

Supplement: Supplementary file 1 [file antioxidants-14-01317-s001.zip › Fig5AB.jpg]

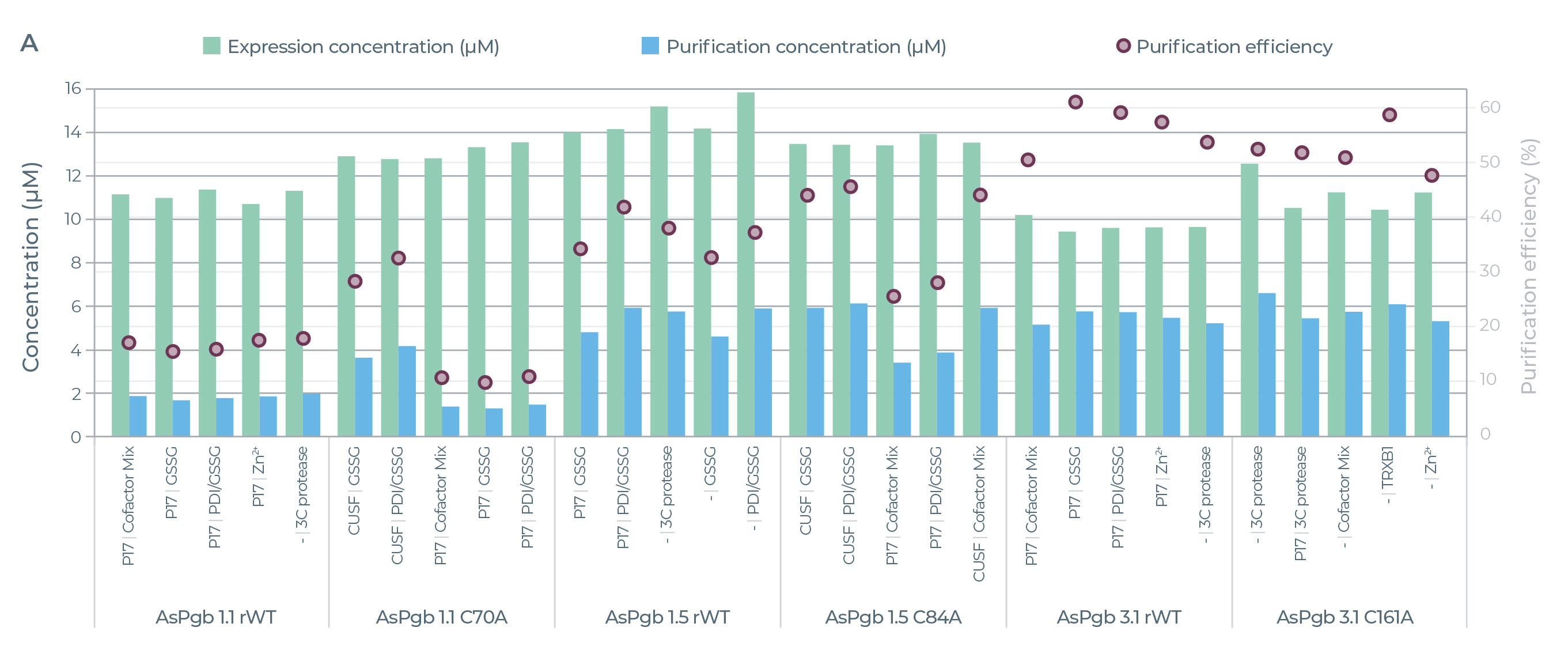

Supplement: Supplementary file 1 [file antioxidants-14-01317-s001.zip › Fig6A.jpg]

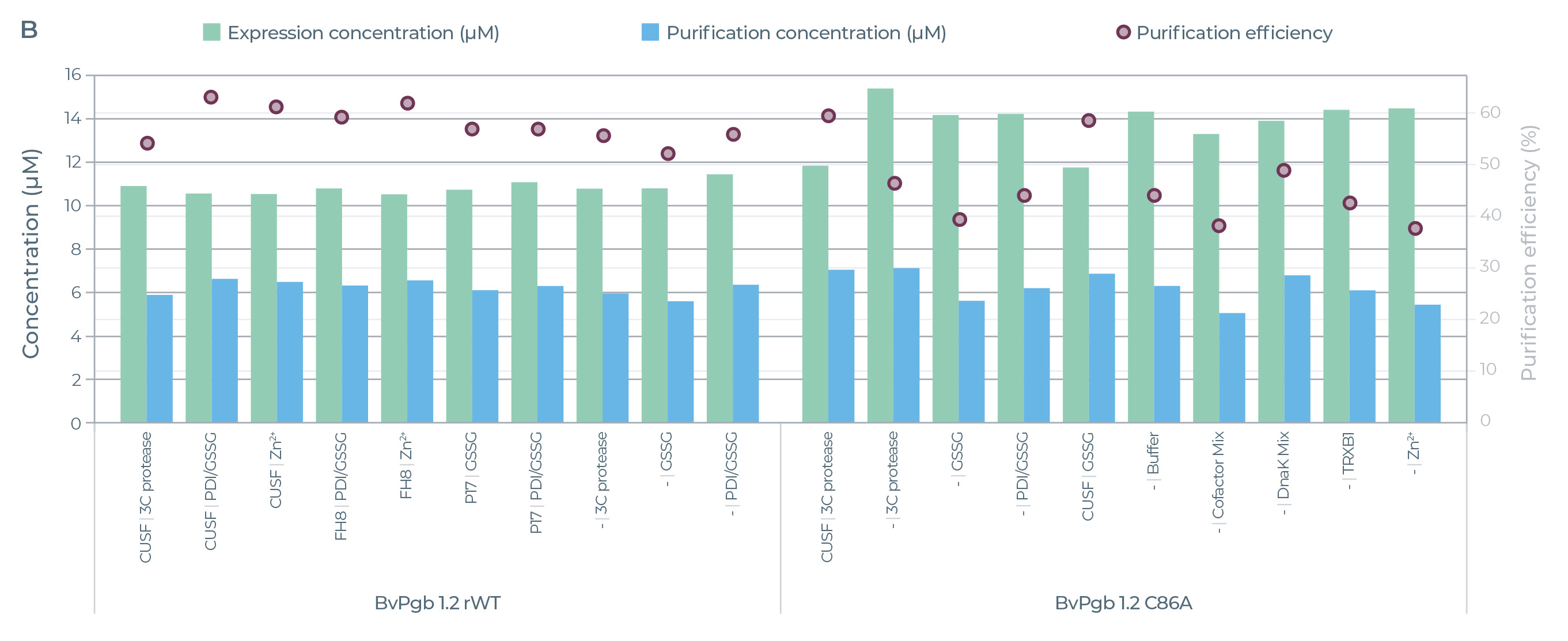

Supplement: Supplementary file 1 [file antioxidants-14-01317-s001.zip › Fig6B.jpg]

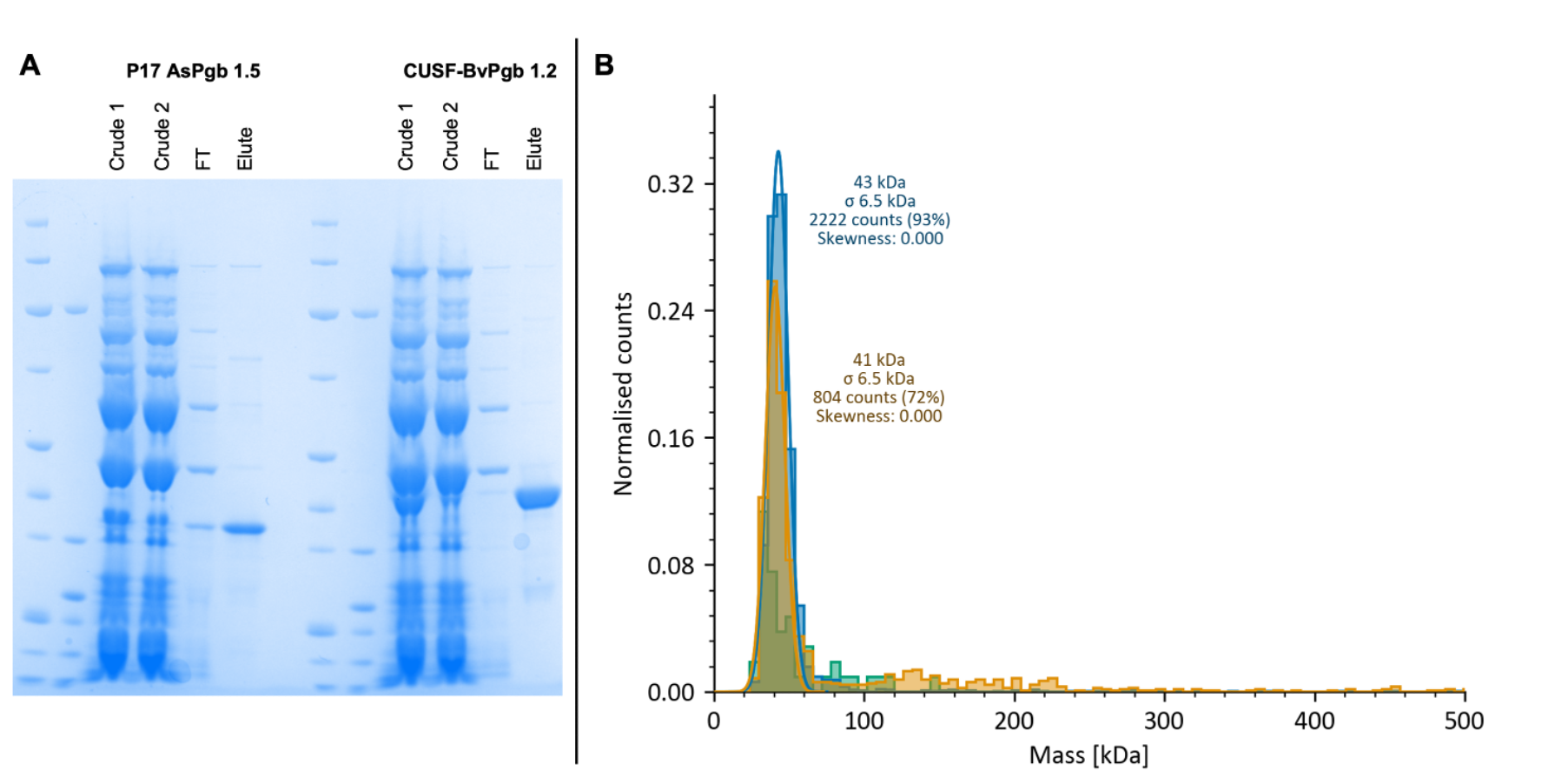

Supplement: Supplementary file 1 [file antioxidants-14-01317-s001.zip › Fig7.png]

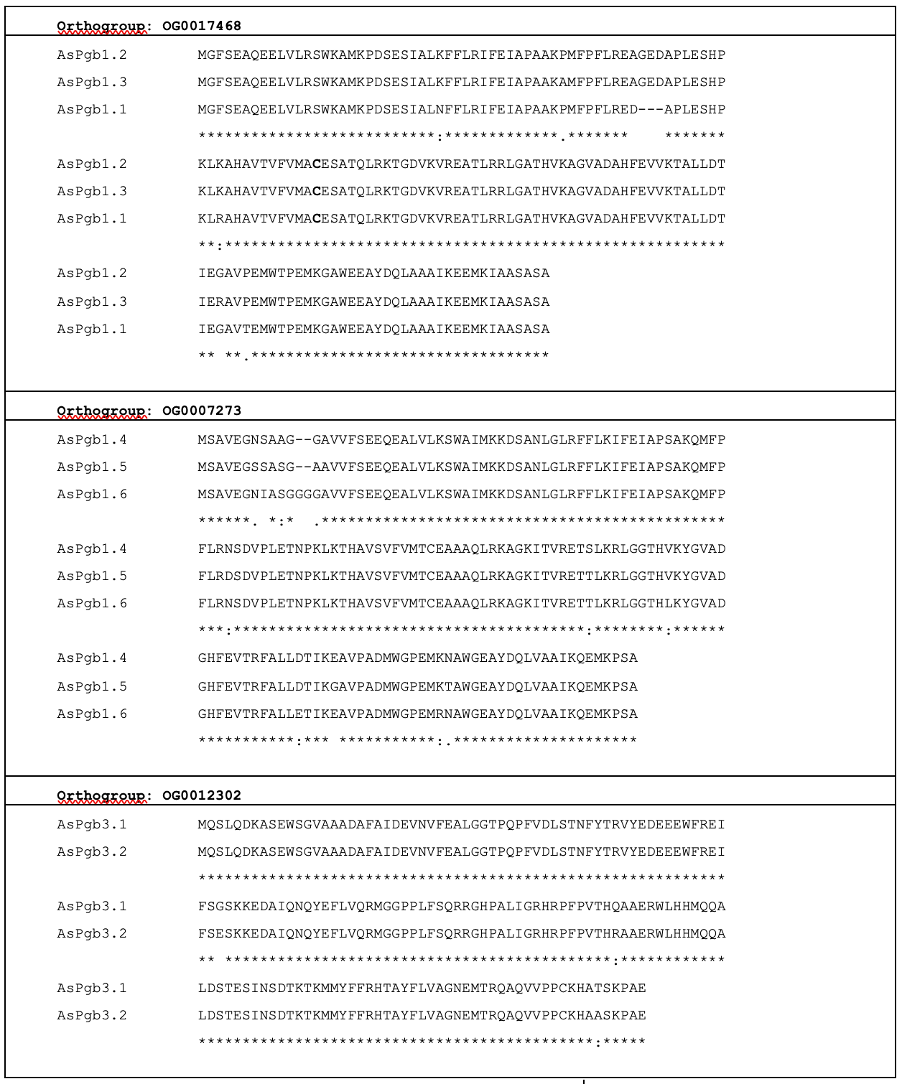

Supplement: Supplementary file 1 [file antioxidants-14-01317-s001.zip › FigS1.png]

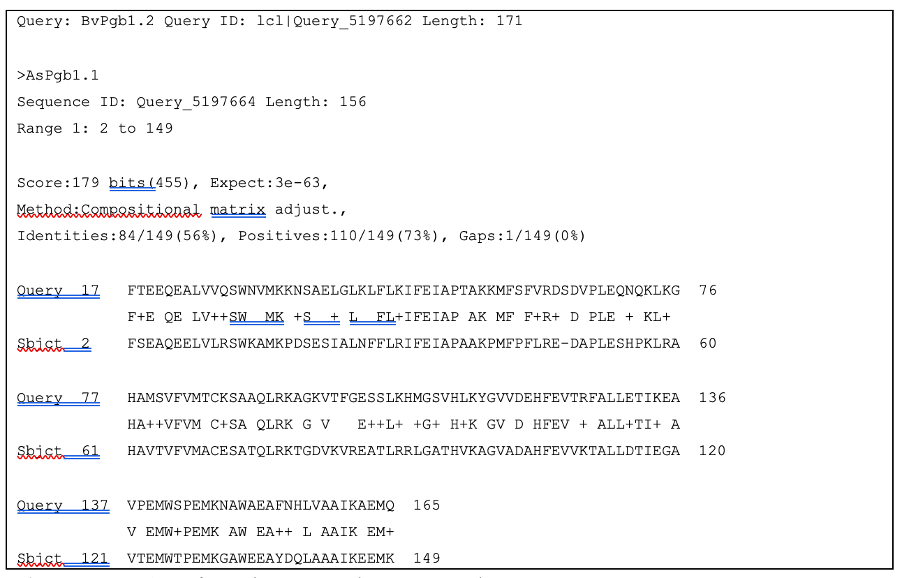

Supplement: Supplementary file 1 [file antioxidants-14-01317-s001.zip › FigS2.png]

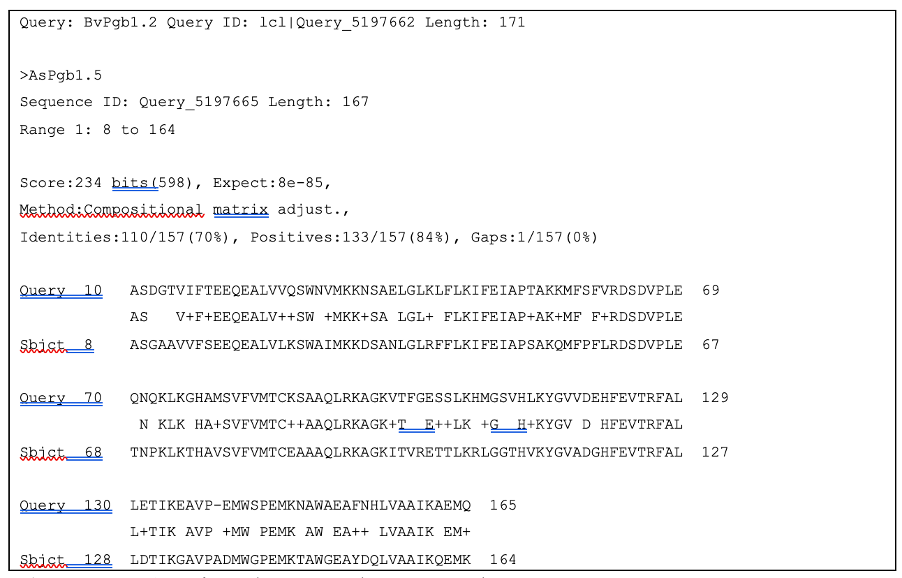

Supplement: Supplementary file 1 [file antioxidants-14-01317-s001.zip › FigS3.png]

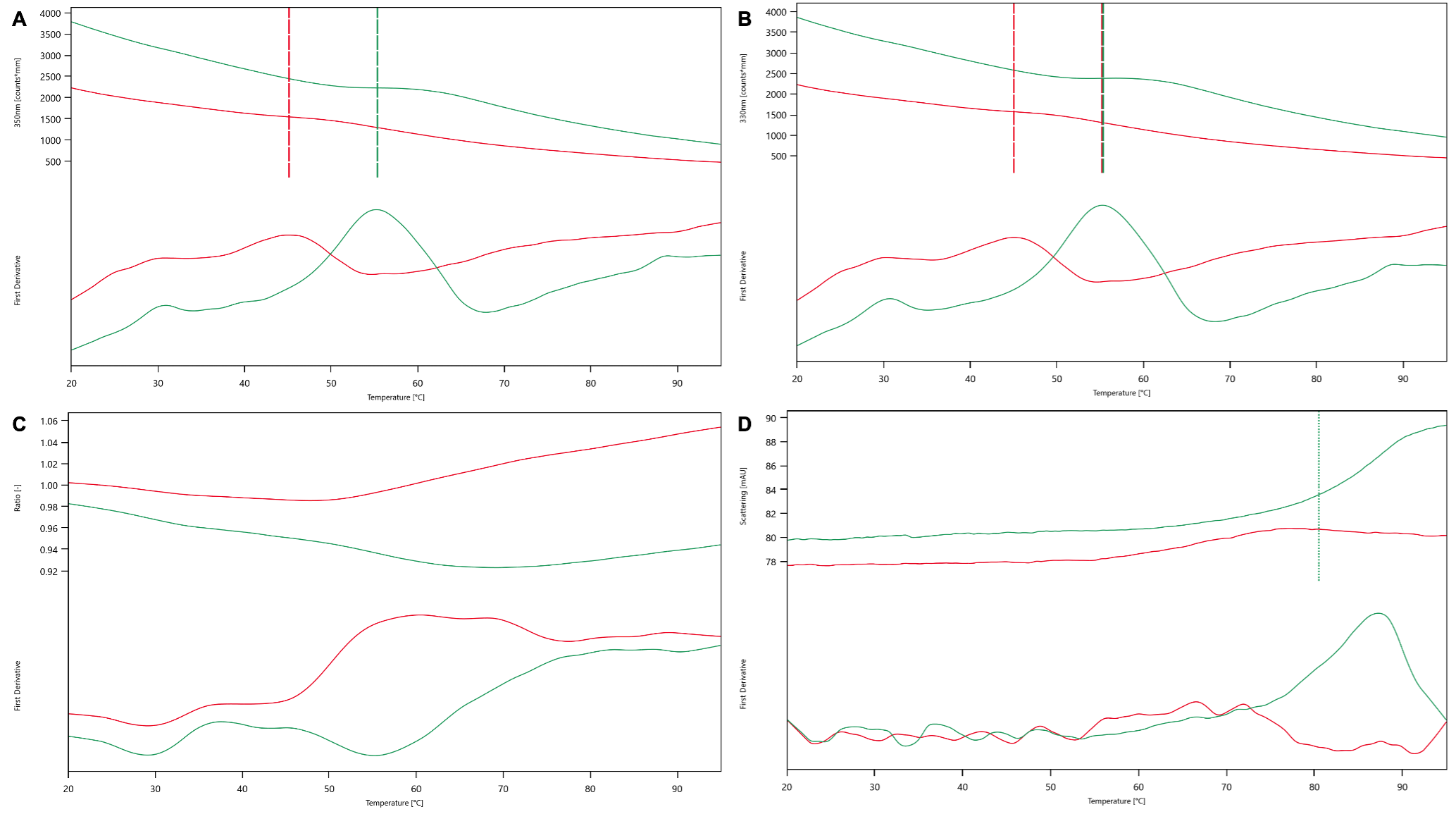

Supplement: Supplementary file 1 [file antioxidants-14-01317-s001.zip › FigS4.png]
